# Assessment of NanoString technology as a tool for profiling circulating miRNA in maternal blood during pregnancy

**DOI:** 10.20517/evcna.2024.38

**Published:** 2024-09-05

**Authors:** Petra Adamova, Andrew K. Powell, Iain M. Dykes

**Affiliations:** ^1^Department of Pharmacy and Biomolecular Sciences, Liverpool John Moores University, Liverpool L3 3AF, UK.; ^2^Liverpool Centre for Cardiovascular Science, Institute for Health Research, Liverpool John Moores University, Liverpool L3 3AF, UK.

**Keywords:** Micro RNA, expression profiling, blood biomarker, pregnancy

## Abstract

**Aim:**

Circulating maternal MicroRNA (miRNA) is a promising source of biomarkers for antenatal diagnostics. NanoString nCounter is a popular global screening tool due to its simplicity and ease of use, but there is a lack of standardisation in analysis methods. We examined the effect of user-defined variables upon reported changes in maternal blood miRNA during pregnancy.

**Methods:**

Total RNA was prepared from the maternal blood of pregnant and control rats. miRNA expression was profiled using Nanostring nCounter. Raw count data were processed using nSolver using different combinations of normalisation and background correction methods as well as various background thresholds. A panel of 14 candidates in which changes were supported by multiple analysis workflows was selected for validation by RT-qPCR. We then reverse-engineered the nSolver analysis to gain further insight.

**Results:**

Thirty-one putative differentially expressed miRNAs were identified by nSolver. However, each analysis workflow produced a different set of reported biomarkers and none of them was common to all analysis methods. Four miRNAs with known roles in pregnancy (miR-183, miR-196c, miR-431, miR-450a) were validated. No single nSolver analysis workflow could successfully identify all four validated changes. Reverse engineering revealed errors in nSolver data processing which compound the inherent problems associated with background correction and normalisation.

**Conclusion:**

Our results suggest that user-defined variables greatly influence the output of the assay. This highlights the need for standardised nSolver data analysis methods and detailed reporting of these methods. We suggest that investigators in the future should not rely on a single analysis method to identify changes and should always validate screening results.

## INTRODUCTION

As many as 6% of births globally are affected by congenital disease^[1]^. The most prevalent of these is congenital heart disease (CHD), which affects 0.8% of newborns (in the UK)^[2,3]^ and accounts for 10% of all infant deaths^[4]^. Antenatal detection improves clinical outcomes for these patients^[5]^, yet current ultrasound-based screening programmes are limited by imprecision^[5]^ and poor detection rates^[6,7]^.

MicroRNAs (miRNAs) are a family of small, non-coding RNAs that function in the post-transcriptional regulation of gene expression^[8]^. miRNA can be transported in the blood as a component of endocrine signalling mechanisms and is delivered to recipient cells either by trafficking within the cargo of extracellular vesicles or by protein carrier mechanisms^[9]^. Changes in levels of expression of miRNA circulating within maternal blood have been reported in a range of congenital diseases including CHD^[10,11]^, Down’s Syndrome^[12-14]^, foetal alcohol spectrum disorders^[15]^, preeclampsia^[16]^, small-for-gestational-age^[17]^ and pre-term birth^[18]^. Extracellular vesicles have been shown to be transferred between foetal and maternal circulations across the placenta in rodent models^[19-21]^, while clinical studies have demonstrated the presence of placental vesicle biomarkers in maternal blood^[22]^ and changes in diseases such as preeclampsia^[23]^. Thus, circulating miRNA offers much promise as a biomarker for the development of non-invasive diagnostic tests for congenital disease. To achieve this potential, robust screening methods must be developed to overcome unresolved issues regarding reproducibility and accuracy^[24-26]^.

There are currently three methods by which to perform a global assay of miRNA expression: amplification-based methods, sequencing-based methods, and hybridisation-based methods^[27]^. Amplification-based methods, such as RT-qPCR, have the highest sensitivity^[27]^ but are expensive and labour-intensive, making them generally inappropriate for global screening. Furthermore, amplification itself can introduce a source of error. Sequencing-based methods (RNA-seq)^[28]^ provide an unbiased global screening tool, but this method is limited by the requirement for extensive bioinformatics processing and analysis of the raw data, which requires specialist training. Hybridisation-based assays such as Nanostring nCounter have the advantage of offering a simple, easy-to-use system for screening a relatively large but limited subset of miRNA (420 for rat, 827 for human)^[29,30]^. The amplification-free preparation, straightforward count-based output, and the ability to process data without the need for specialist bioinformatics training using proprietary software make the system attractive as a global screening tool for biomarker discovery.

A number of studies have used Nanostring nCounter to profile circulating maternal miRNA expression, but the field suffers from a lack of consistency in data processing methodology^[14,17,31-40]^. Although the process is automated by Nanostring’s proprietary nSolver software, a number of user-defined variables must be set. These include the method used to adjust for background correction, the stringency of this background correction and the method used to normalise samples. Commonly, researchers do not justify their choice of a given analysis method, while in some cases, details of the analysis methods are not provided in enough detail to replicate. As we demonstrate below, these variables greatly influence the output of the system.

Analysis of circulating miRNA is complicated by two factors. Firstly, all assays produce a low level of noise, yet the low expression levels of many miRNAs in blood present a problem in distinguishing signals from noise. If the threshold is too high, then the real signal is lost, generating false negatives, while a threshold that is too low results in false positives due to genuine noise. nSolver offers two different methods by which background correction can be performed: background thresholding and background subtraction^[41]^. Background thresholding is the process of adjusting all counts that are below the calculated background level to bring them up to this threshold. Background subtraction is the process of reducing all counts by the background level. This latter is a useful technique when the estimates of counted transcripts above background noise are a key finding. However, background subtraction can result in overestimation of fold changes in low-expressing targets^[42]^.

A second problem is a lack of established endogenous normalisers expressed at a stable level. In tissue samples, other species of small RNA with housekeeping functions such as mRNA splicing factors may be used, but these are not present in blood. This presents a unique challenge in the analysis of blood miRNA, necessitating either the use of an exogenous spike-in, identifying such stable endogenous normalisers within the test sample itself or performing a global averaging across all expressed miRNA. NanoString recommends using one of two methods for normalisation: these essentially involve normalising to a global average of all targets expressed above threshold in all samples (referred to as the total RNA method) or alternatively by identifying a small cohort of the most stably expressed targets to use as normalisers (referred to as the NormFinder method)^[43]^.

In order to explore this problem, we designed a study in which we examined the ability of nCounter to detect changes in circulating miRNA between pregnant and control conditions in blood from rats. We identified key user-defined variables within the nSolver data processing workflow, which differed between published protocols, and examined the effect of each on the detected changes. Following validation of four changed miRNA by RT-qPCR, we then reverse-engineered the system to look in detail at the data processing steps performed by nSolver. Our results suggest that a single analysis workflow, as has been used in most published studies to date, is insufficient to identify all changes. We propose a method in which results from a number of analysis workflows are integrated.

## METHODS

### Blood collection from pregnant and control rats

The protocol for animal work described in this study was reviewed and approved by the Liverpool John Moores University Animal Welfare and Ethics Review Board in January 2020 (Ref: ID_PA/2023-3). Twelve Wistar female rats were divided into a control and a pregnant group of equal size (*n* = 6). The oestrous cycle of the pregnant group was monitored using daily vaginal smears. 100 µL of PBS containing the vaginal cell suspension was air-dried on a glass slide, stained with 0.1% Toluidine Blue and examined under a light microscope to determine the relative proportions of cell types. Proestrus was identified by high proportions of nucleated epithelial cells. Females in proestrus were paired overnight with a male. Pregnancy was confirmed by the presence of a mucosal plug in the cage and the presence of sperm in the vaginal smear. Pregnant rats were sacrificed at midday 14 days post mating (day E14.5) by Schedule 1 methods (CO_2_ inhalation followed by cervical dislocation to ensure death). Blood samples were taken by post-mortem cardiac puncture performed immediately after confirmation of death using a 22-gauge needle inserted 5 mm from the centre of the thorax, angled towards the animal’s chin and pushed 5-10 mm deep to penetrate the heart. Whole blood was withdrawn into 6 mL Ethylenediaminetetraacetic acid-treated tubes (Vacutest Kima, Arzergrande, Italy). Pregnancy was confirmed following blood collection by examination of the uterus. Blood was taken from age-matched non-pregnant controls by the same method.

### Preparation of platelet-free plasma

Whole rat blood was immediately centrifuged at 3,000 rpm (1,811 rcf) for 30 min at room temperature in an Eppendorf Centrifuge 5,810 R to pellet cells. The top layer (plasma) was then transferred into RNase-free 1.5 mL microcentrifuge tubes and centrifuged at 13,000 rpm (14,549 rcf) for 30 min at 4 °C in a benchtop Eppendorf Centrifuge 5,418 to remove platelets. The supernatant was centrifuged a second time at 13,000 rpm (14,549 rcf) for 5 min at 4 °C to ensure complete platelet removal and the supernatant was then transferred to a fresh tube. Plasma was stored at -80 °C.

### Total RNA preparation

RNA was prepared using the method recommended by Nanostring^[43]^. A total of 1.5 mL of plasma was used. Total RNA was prepared using the Plasma/Serum RNA Purification Midi Kit (Norgen Biotek, Ontario, Canada) following the manufacturer’s protocol. 5 µL of a 200 pM solution of an exogenous spike-in miRNA control from rice (osa-miR-414) was added to each sample at the start of the protocol. Following purification, the volume of the sample was reduced to 20 µL using an Amicon Ultra-0.5 mL centrifugal filter (3 kDa cutoff) (Merck, Cork, Ireland). RNA samples were then stored at -80 °C.

### Haemolysis control

Haemolysis was monitored prior to nCounter profiling by performing RT-qPCR (below) for the erythrocyte-specific miRNA miR-451a and the stably expressed miRNA, miR-23a. Non-haemolysed blood should have a 451/23 ratio < 5.

### Nanostring nCounter miRNA profiling

Five µL of the total RNA preparation was used for nCounter profiling. Ligation of miRtags, hybridisation to probes, washing, immobilisation to solid substrate, scanning and data collection were performed by Liverpool University Centre for Genomic Medicine following the manufacturer’s recommendations. The Rat v1.5 miRNA codeset (Nanostring Technologies, Seattle, USA) was used for this analysis. Raw data have been uploaded to the Gene Expression Omnibus (GEO accession number: GSE267016).

### nSolver miRNA data analysis

Raw count data obtained from nCounter were processed to obtain a list of potential changed miRNA using nSolver Analysis v4.0 (Nanostring Technologies, Seattle, USA). As described in the Results section, the analysis was performed using various combinations of normalisation method, background correction, and background levels.

### Identification of control normalisers within the dataset

To identify a set of miRNA to be used for global normalisation with the “total miRNA” method, we used nSolver software. No positive control normalisation was performed and the background was set to the mean plus 1 standard deviation (mean + 1 SD) of the negative control probes. This value was subtracted from the raw count value. Any probe with expression above this threshold in all 12 rats was included in the “total miRNA” pool. To identify miRNA to use as normalisers for the NormFinder method, we used the NormFinder Excel plug-in^[44]^ to identify the five most stably expressed (lowest variance) from the total miRNA pool. The two most stable miRNAs identified by Normfinder were used as normalisers for the validation of RT-qPCR.

### Multivariate analysis

Principal component analysis (PCA) was performed on the 14 analysis workflows using the R Shiny opensource package (https://mikies21.shinyapps.io/shinybeetlenmr/) with the aim to compare the global plasma miRNA expression of the pregnant rats to that of the non-pregnant controls.

### RT-qPCR

Reverse transcription and SYBR green-based quantitative PCR were performed using the miRcury Locked nucleic acid (LNA) miRNA PCR Assay system (Qiagen, Manchester, UK). A UniSp6 spike-in control (0.075 fmoles) was added prior to reverse transcription. 0.5 µL (equivalent to 40 µL plasma) of the 20 µL total RNA preparation was reverse-transcribed in a volume of 10 µL. During reverse transcription, a poly(A) tail is ligated to the miRNA and cDNA is synthesized using a Poly(T) primer with a 3’ degenerate anchor and a 5’ universal tag. The resulting cDNA was diluted 1:30 in water and 3 µL used for each qPCR reaction. This cDNA template was then amplified using two miRNA-specific primers [Table 1] which contain locked nucleic acid providing specificity down to 1 nucleotide. Quality control steps were performed on a Rotor-Gene Q real-time cycler (Qiagen). Thresholds were set in the exponential phase of the amplification plot at 0.01 fluorescence (ΔRn) for all miRNA targets. Technical validation of candidate plasma miRNAs was performed on an Applied Biosystems 7,500 machine using ROX as a passive reference dye. Thresholds for each miRNA used to determine the quantification cycle (Cq) values were automatically set in the exponential phase of the amplification plot and baselines were manually positioned at approximately 2 cycles prior to the first visible amplification. The raw Cq values were normalised to two stably expressed endogenous normalisers identified using the NormFinder algorithm (miR-20a, miR-27b).

**Table 1:** Primers used in miRcury RT-qPCR assays

**Functio*n***	**miRNA**	**miRbase accession**	**Qiagen assay number**	**miRNA Sequence (5’-3’)**
Spike-ins	osa-miR-414	MIMAT0001330	ZP00007870	UCAUCCUCAUCAUCAUCGUCC
	UniSp6	*n*/a		Proprietary
RNA Purification QC	hsa-miR-191-5P	MIMAT0000440	ZP00000368	CAACGGAAUCCCAAAAGCAGCUG
	hsa-miR-103-3p	MIMAT0000101	YP00204063	AGCAGCAUUGUACAGGGCUAUGA
	hsa-miR-23a-3p	MIMAT0000078	ZP00000478	AUCACAUUGCCAGGGAUUUCC
Haemolysis QC	hsa-miR-451a	MI0001729	ZP00001151	AAACCGUUACCAUUACUGAGUU
Normalisers	hsa-miR-20a-5p	MIMAT0000075	YP00204292	UAAAGUGCUUAUAGUGCAGGUAG
	hsa-miR-27b-3p	MIMAT0000419	YP00205915	UUCACAGUGGCUAAGUUCUGC
Test probes	hsa-let-7b-5p	MIMAT0000063	YP00204750	UGAGGUAGUAGGUUGUGUGGUU
	hsa-let-7d-5p	MIMAT0000065	YP00204124	AGAGGUAGUAGGUUGCAUAGUU
	hsa-miR-125a-5p	MIMAT0000443	YP00204339	UCCCUGAGACCCUUUAACCUGUGA
	hsa-miR-132-3p	MIMAT0000838	YP00206035	UAACAGUCUACAGCCAUGGUCG
	hsa-miR-133a-3p	MIMAT0000427	YP00204788	UUUGGUCCCCUUCAACCAGCUG
	hsa-miR-183-5p	MIMAT0000261	YP00206030	UAUGGCACUGGUAGAAUUCACU
	hsa-miR-423-3p	MIMAT0005313	YP00204488	AGCUCGGUCUGAGGCCCCUCAGU
	hsa-miR-431-5p	MIMAT0001625	YP00204737	UGUCUUGCAGGCCGUCAUGCA
	mmu-miR-872-5p	MIMAT0005282	YP00205481	AAGGUUACUUGUUAGUUCAGG
	rno-miR-1224	MIMAT0012827	Custom	GUGAGGACUGGGGAGGUGGAG
	rno-miR-196c-5p	MIMAT0005303	Custom	UAGGUAGUUUCGUGUUGUUGGG
	rno-miR-3563-5p (rno-mir-299b)	MIMAT0017833	Custom	CGGUUUACCGUCCCACAUAC
	rno-miR-3573-5p	MIMAT0017856	Custom	UGAGGGGCAGUGAUAGAAAGGA
	rno-miR-450a	MIMAT0001547	Custom	UUUUGCGAUGUGUUCCUAAUGU

Note that in some cases, the human (hsa) or mouse (mmu) miRNA has an identical sequence to that in the rat (rno), and therefore, these primers may be used.

### *In silico* reanalysis of nSolver data

To further understand NanoString nCounter Technology, a recalculation was performed using Microsoft Excel. Following the information provided in the NanoString tech notes^[42]^, recalculation of background correction, positive control normalisation, and content normalisation was performed. It was possible to obtain nSolver outputs for intermediate steps in the analysis, and in this way, it was possible to carefully match these recalculated values to the nSolver outputs. This enabled the correct order of steps performed by the nCounter analysis software to be identified. Once the correct order was identified, the different workflows were recalculated for the final 3 validated miRNA targets.

### Statistics

Nanostring nCounter compares the final calculated expression values between groups using Welch’s *t*-test. To mimic nSolver, a Welch’s *t*-test was also used for the *in-silico* recalculation analysis. For RT-qPCR validation of candidate changes, data were tested for normality using the Shapiro-Wilk test. For normally distributed data, an unpaired Student’s *t*-test was used to compare two groups, while for non-parametric results, a Mann-Whitney *U* test was used. In all tests, a *P*-value of < 0.05 was considered to be statistically significant.

## RESULTS

### Experimental design to evaluate Nanostring nCounter analysis

**Figure 1:**
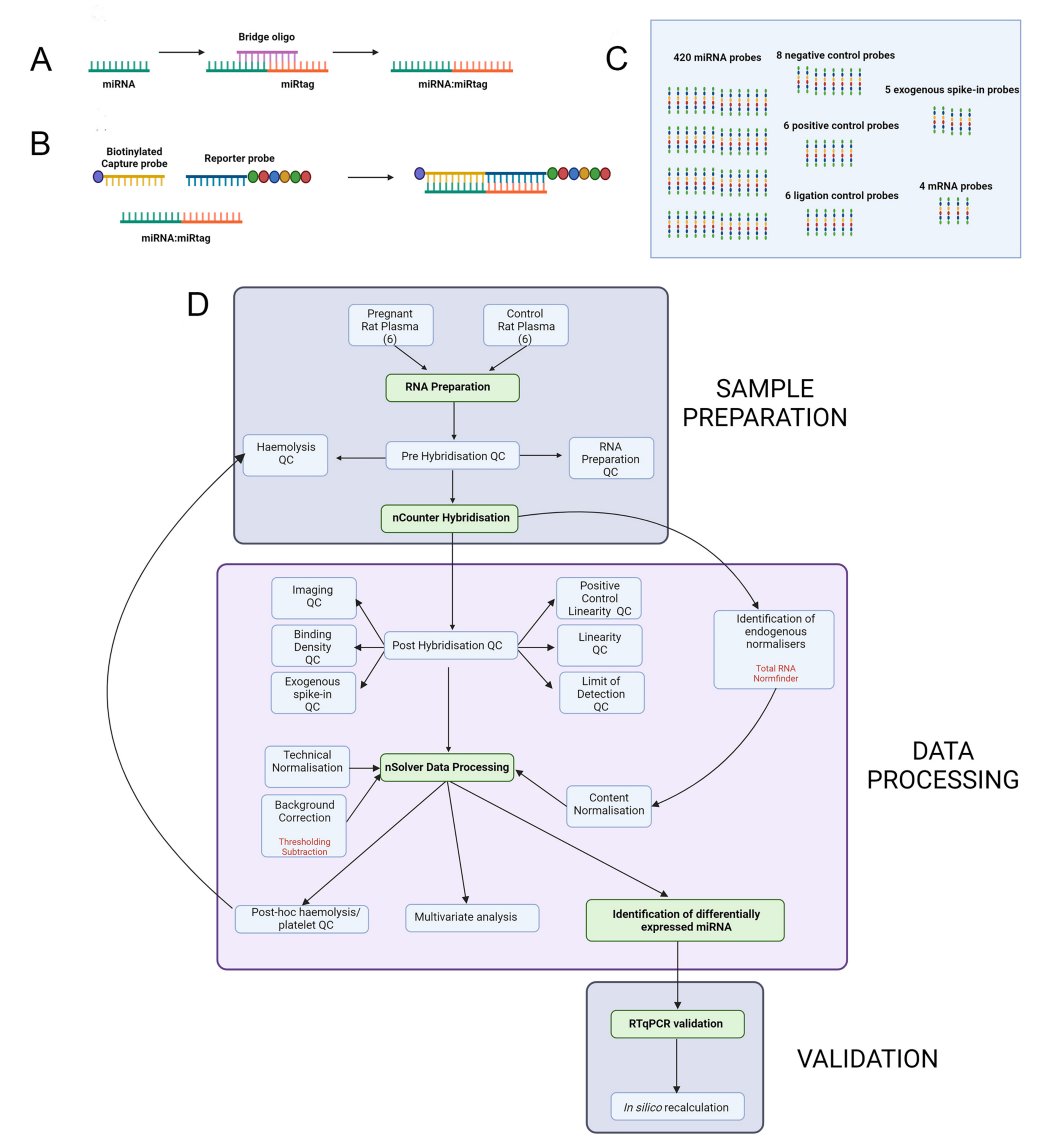
Overview of the experiment. miRNA: MicroRNA; A and B: principle of the Nanostring nCounter assay; A: miRNAs (green) are too short for standard probe attachment. To overcome this, a temporary bridging oligo (purple) is used to extend the length of each miRNA through the attachment of a DNA-based miRtag (orange) by splinted ligation; B: during the hybridisation step, the extended miRNA: miRtag is hybridised to a biotin-tagged capture probe (yellow) and a reporter probe (blue) carrying a unique fluorescent barcode tag (6 coloured circles). After washing, the target/probe complexes are immobilised to a solid substrate using the biotin tag and data recorded from the barcode as individual counts; C: set-up of the rat v1.5 miRNA codeset, which contains probes for 420 rat miRNAs as well as positive, negative and ligation controls, exogenous spike-in controls and housekeeping mRNA probes; D: flowchart to illustrate the workflow described in this paper. Figure made with Biorender.

The Nanostring nCounter is a hybridisation-based assay in which a panel of fluorescently-labelled probes is used to assay miRNA expression [Figure 1A and B]. The codeset consists of test probes for 420 endogenous rat miRNA (827 in the human assay), together with a number of control probes [Figure 1C], the function of which will be described below. The raw output of a nCounter assay consists of counts for each probe. These raw counts are processed using nSolver software with user-selected variables that vary according to the study [Figure 1D].

We tested the workflow of NanoString miRNA analysis using a rat pregnancy scenario. Rats were chosen as our animal model of choice due to their short gestation time and the ability to obtain sufficient blood for analysis. We used a 6 × 6 experimental design in which 6 plasma samples from pregnant rats were compared to 6 controls. This number was chosen because 12 samples can be run together on a single nCounter cartridge, reducing technical variance.

In the sample preparation phase [Figure 1D upper box], timed rat matings were performed, maternal blood was collected, and total RNA was prepared from platelet-free plasma. Pre-hybridisation quality control was performed by RT-qPCR for RNA preparation and to verify that the level of haemolysis was below an acceptable threshold.

Data processing following hybridisation to the nCounter is a key step [Figure 1D middle box]. A number of post-hybridisation quality control metrics were examined to verify successful hybridisation. A set of endogenous normalisers were identified within the dataset using two methods - the total RNA and Normfinder methods. Raw count data were then processed using nSolver. As described below, this process involves a number of user-defined steps including background correction (by either thresholding or subtraction) at a user-defined background level. Two normalisation steps are performed: the first is a technical normalisation to control for different hybridisation efficiencies within the assay, while the second content normalisation utilises the total RNA and Normfinder endogenous normalisers. Following processing, we performed some post-hoc quality control assays for haemolysis and platelet contamination. We then performed both multivariate analysis to look at global trends in the data and univariate analysis to identify specific miRNA differentially expressed between conditions.

In the validation phase [Figure 1D], we first validated candidate changed miRNAs by RT-qPCR before lastly performing an *in silico* analysis of validated miRNA to understand precisely how these changes were identified by the software.

### Rat mating and RNA preparation

A pregnant group of 6 rats of various ages was compared to an age-matched control group of 6 rats [Table 2 and Figure 1 upper box]. Each cohort consisted of 4 younger rats (103-112 days old) and two older rats (239-373 days).

**Table 2:** Animals used in this study

**Pregnant group**				**Control group**	
**Rat code**	Age at blood collection (days)	Weight on day 0.5 (g)	Weight gain by day 14.5 (%)	**Rat code**	Age at blood collection (days)
**R11**	103	261	34	**R5**	239
**R14**	104	267	21	**R8**	269
**R9**	269	263	25	**R20**	110
**R12**	373	361	15	**R22**	111
**R18**	110	259	18	**R23**	112
**R19**	110	265	25	**R24**	112
**Mean**	193	279	23	**Mean**	159
**SD**	122	40	7	**SD**	74

**Figure 2:**
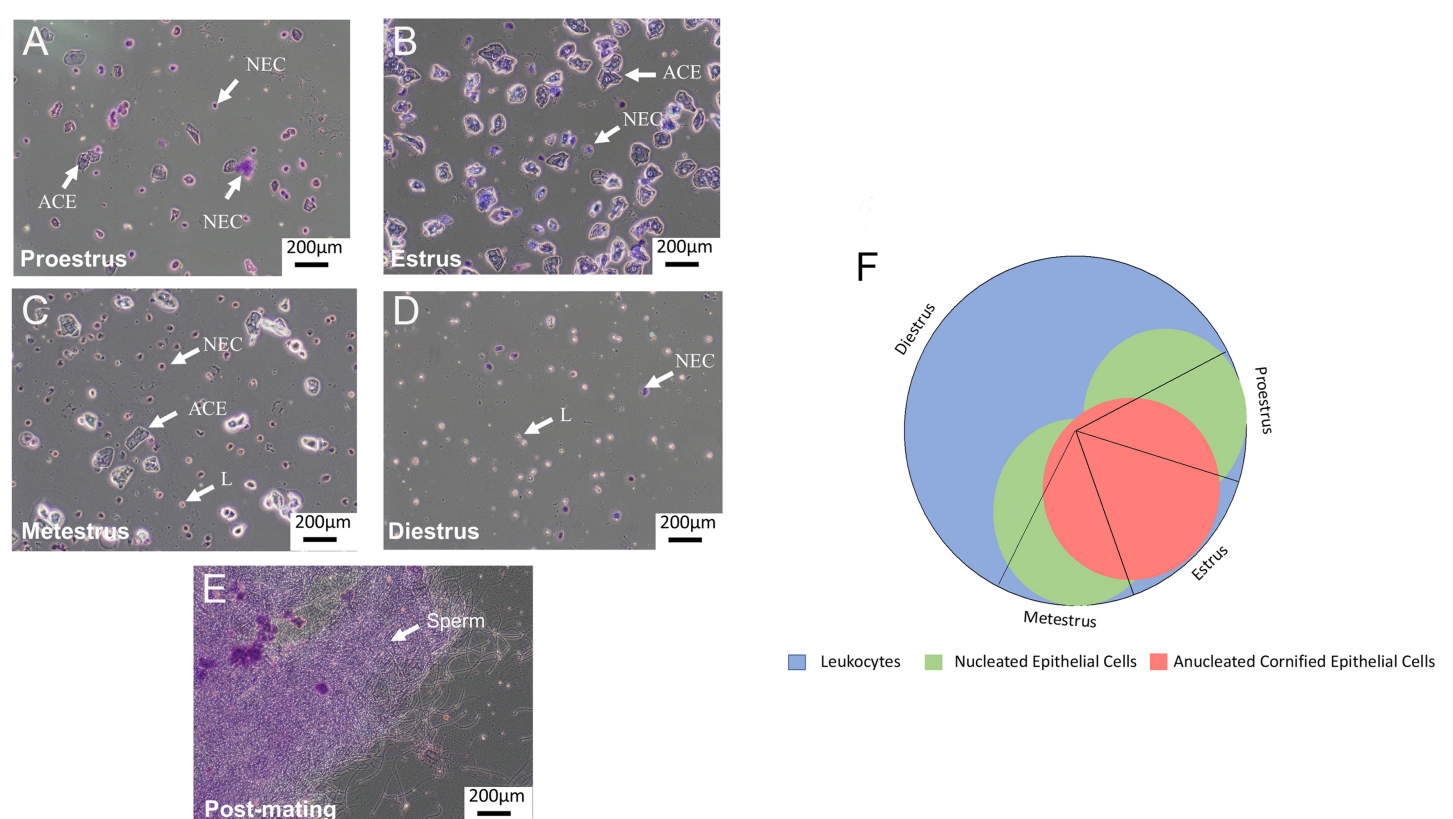
Rat oestrus cycle and mating monitoring. ACE: Anucleated cornified epithelial cell; NEC: nucleated epithelial cell; L: leukocyte; A-E: images show rat vaginal smears stained with toluidine blue to label DNA; F: graphical representation of the 4-5 day long rat oestrus cycle representing the proportions of cell types present in each phase. F is adapted from^[45]^.

Timed matings were set up [Figure 2] and post-mortem blood was taken at embryonic day E14.5 from the pregnant group and matched controls and total RNA was prepared from 1.5mL platelet-free plasma using the method recommended by NanoString for assaying circulating miRNA in plasma^[43]^.

### Pre-hybridisation quality control

It is not possible to quantify plasma RNA using a standard spectrophotometer because it is below the level of detection. Therefore, to validate successful RNA preparation prior to screening [Figure 1D upper box], quality control was performed by RT-qPCR using a panel of probes. The chosen probes included primers for two exogenous spike-ins (osa-miR-414, added to the RNA preparations and UniSP6, added during reverse transcription) and 3 primers for endogenous miRNAs previously reported to be strongly expressed in plasma (miR-191-5p, miR-103-3p, miR-23a-3p)^[46,47]^.

**Figure 3:**
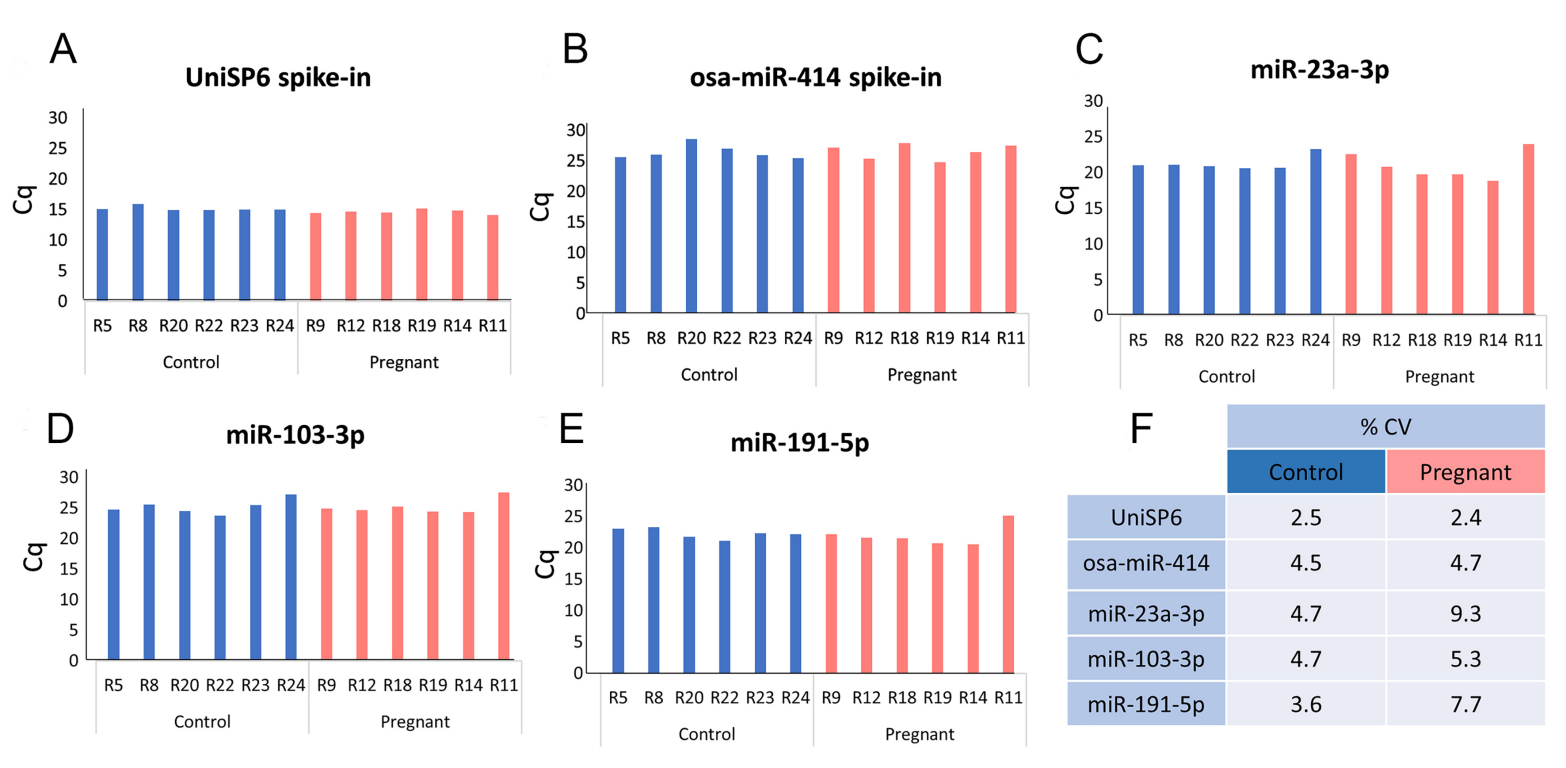
RNA preparation quality control - detection of spike in controls and stably expressed plasma miRNA. Thresholds were set in the exponential phase of amplification (0.01 ΔRn) and the same threshold was used for each miRNA. miRNA: MicroRNA; A-E: graphs show the quantification cycle (Cq) of the indicated miRNA for each of the 12 rats; F: table to show the percentage coefficient of variance of Cq value for each probe within each group. Single technical replicate per rat due to limited sample availability, 6 biological replicates per group as show.

All miRNAs were detected in all 12 samples, demonstrating successful RNA preparation and successful reverse transcription [Figure 3]. As expected, the reverse transcription spike-in UniSp6 gave the lowest Cq values [Figure 3A], indicating the highest expression levels, while the osa-miR-414 spike-in gave the highest Cq values [Figure 3B]. The two spike-ins also had the lowest variance within each group and this variance was largely consistent between groups [Figure 3F]. The three endogenous miRNAs tested had the highest variance, and in all cases, the variance was higher in the pregnant than in the control group [Figure 3C-F].

**Figure 4:**
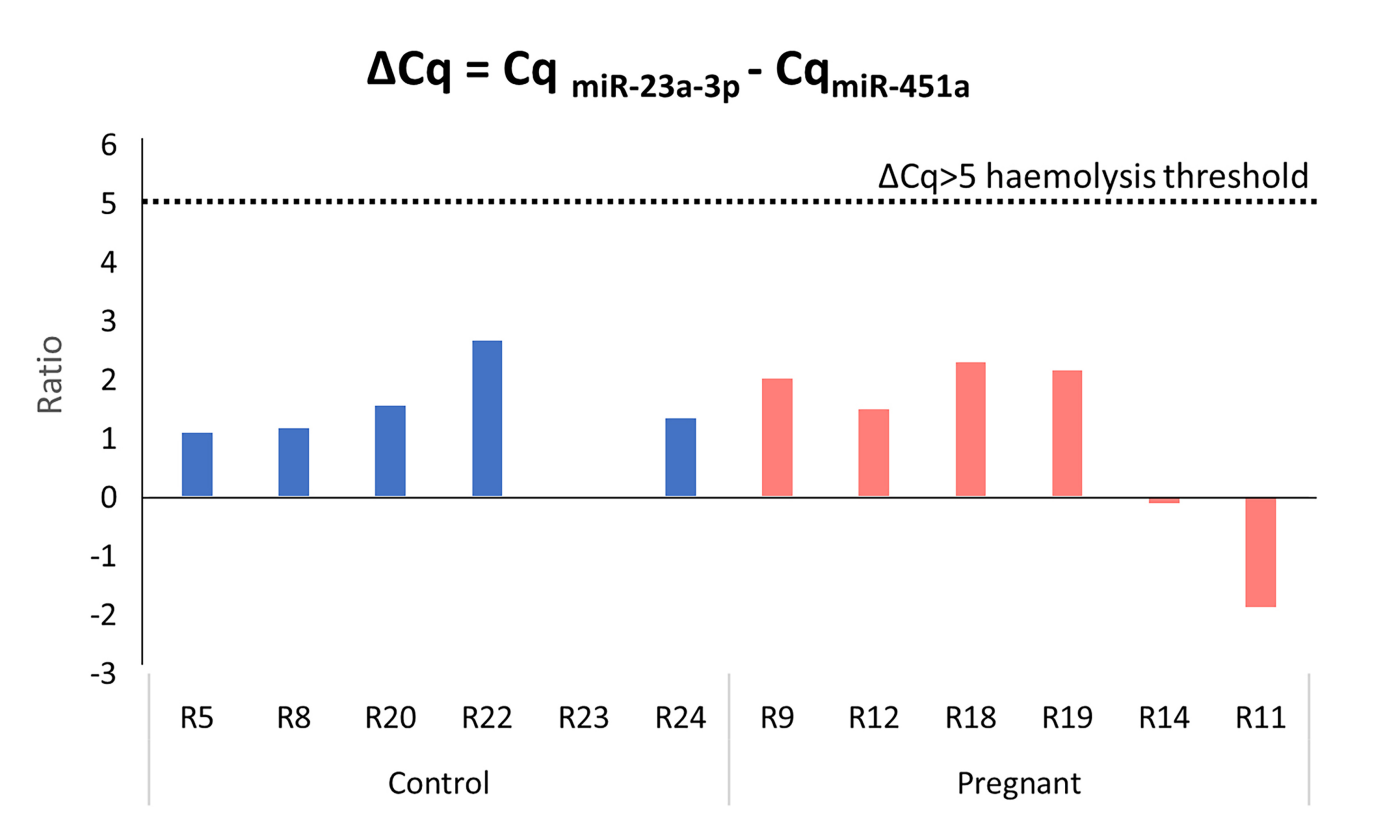
Haemolysis analysis quality control. The graph shows the ratio of miR-23-3p expression divided by miR-451a expression calculated from the difference in Cq values. Cq: Quantification cycle. The threshold indicates a ratio of 5, which is considered to indicate haemolysis. Single technical replicate per rat due to limited sample availability, 6 biological replicates per group as shown. Thus, sample preparation [Figure 1D upper box] was successful and all samples passed quality control.

Red blood cells are known to carry specific miRNAs, and therefore, any haemolysis during sample preparation can alter miRNA expression in samples, masking any real difference between groups^[47-49]^. To monitor the level of haemolysis of the 12 samples, we used a miRNA-based method developed by Blondal *et al.* at Exiqon^[47]^, which has been shown to be more sensitive than haemoglobin-based analysis methods with a sensitivity down to 0.001% haemolysis^[50]^. Blondal’s method compares the relative expression of the erythrocyte-specific miRNA miR-451a to a stable reference miRNA, miR-23a-3p [Figure 3C], with a ratio greater than 5 fold, indicating likely haemolysis. All samples showed a ratio well below this threshold [Figure 4].

### nCounter assay

Following RNA preparation and quality control, samples were assayed using the Nanostring nCounter with a Rat v1.5 miRNA codeset [Figure 1A-C and Figure 1D upper box].

### Post-hybridisation quality control

The first step in the data processing phase [Figure 1D middle box] was the post-hybridisation quality control. This was performed using readouts from the control probes included in the codeset [Figure 1C]. These metrics indicated no technical issues with nCounter hybridisation [Supplementary Table 1]. Imaging quality control is a measure of the proportion of fields of view for which imaging was successful over those attempted. The recommended minimum threshold is 75%; all of our data obtained a value of 99%. Binding density is a measure of image saturation that can interfere with probe detection. All our samples were within the range of 0.14-0.17, at the lower end of the acceptable saturation range of 0.1-2.25. Positive control linearity quality control (QC) is a regression number calculated from the count data of the positive control probes, which have a known linear concentration range from 128 fM to 0.125 fM. Our samples have a regression value of 0.98-0.99, above the 0.95 threshold. The limit of detection QC compares the count of the positive control probe Pos_F present at 0.5 fM to the count for the negative control probes. This count should be higher than the mean plus two standard deviations (mean + 2 SD) of the negative control probes [Supplementary Table 2] and this was found to be the case for all samples. Similarly, the ligation QC checks that the count for synthetic spike-ins is above the negative control counts.

The assay contains probes for 5 exogenous spike-in miRNA controls [Figure 1C]. We used one of the five in our assay (osa-miR-414), adding this during RNA preparation. osa-miR-414 gave an average count of 20 across all samples in unprocessed data (data not shown). This was expressed above mean + 2 SD of the negative control probes in only 5 of the 12 rats, while in 4 rats, the count was below the negative control mean. Of note, the mean count data for another exogenous miRNA probe (ath-miR159a, for which we did not add a spike-in) gave the same mean count. Thus, we concluded that the osa-miR-414 spike-in was below the level of detection and could not be used to monitor RNA preparation variation. We did not use this in further analysis.

### Effect of background subtraction on probe count

**Figure 5:**
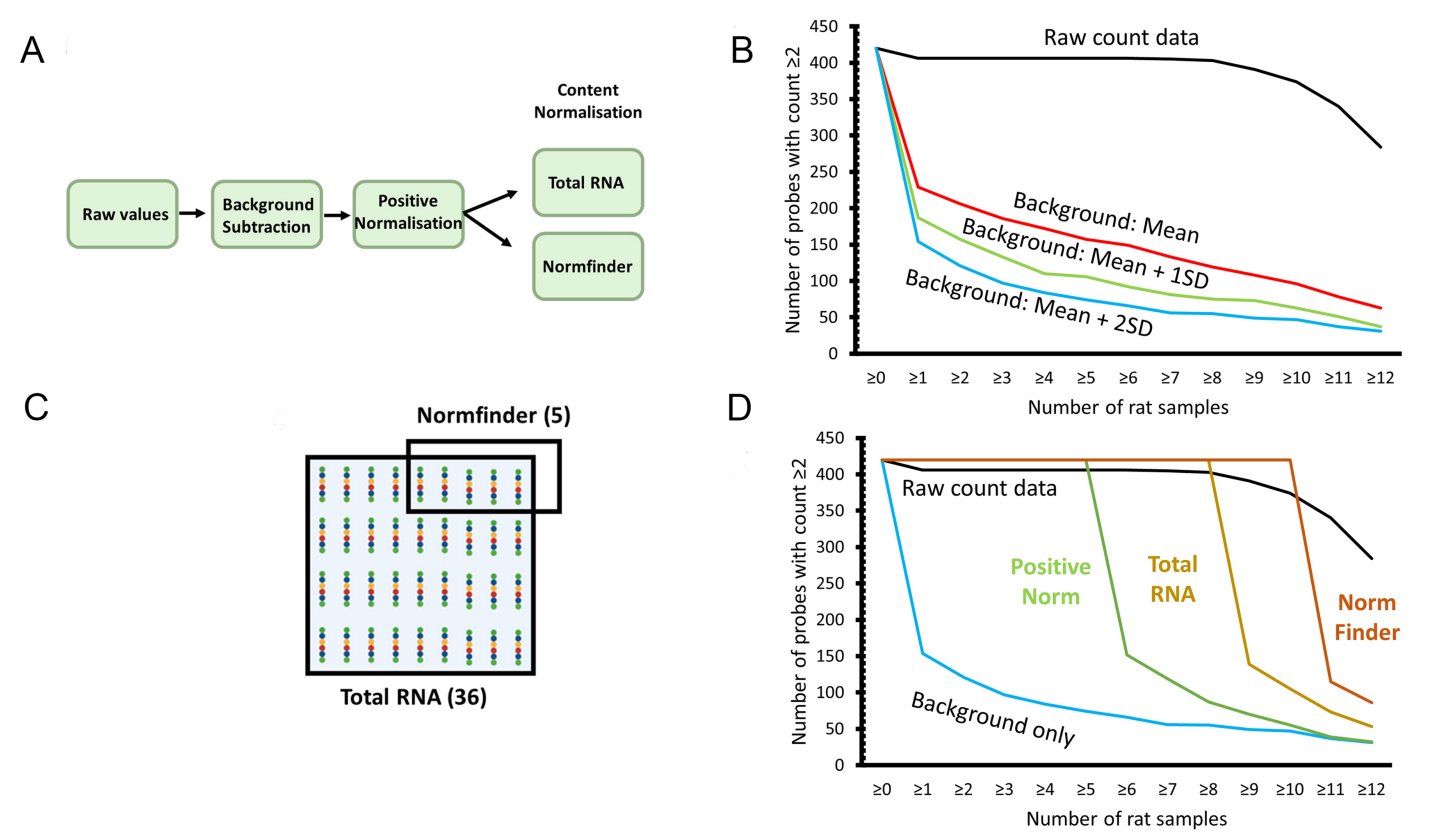
Effect of background correction and normalisation on probe count. SD: Standard deviation; A: flowchart to illustrate the order of data processing in this analysis. Raw count data were first subjected to background subtraction at one of three thresholds (mean, mean + 1 SD, mean + 2SD), they were then normalised using positive control probes before undergoing content normalisation by either the total RNA or Normfinder method; B: effect of background subtraction, the graph shows the number of probes with a count above 2 that were detected in the 12 rat samples. The unprocessed raw count data are shown at the top (black line), followed by processed data using 3 background thresholds (red, blue, green); C: in our analysis, a panel of 36 probes was used as normalisers for the total RNA method, a subset of 5 of the most stable of these was used for Normfinder normalisation; D: effect of normalisation, data plotted as shown in c. The unprocessed raw count data are shown at the top (black line). All processed data use a background threshold of mean + 2 SD (blue). Green line shows the effect of applying positive control normalisation to the background subtracted data, while gold and orange lines show the effect upon these data of subsequent content normalisation by total RNA and Normfinder methods, respectively.

Before performing the main analysis to identify differentially expressed miRNA, we first wanted to understand the effect of user-defined data processing options within nSolver software [Figure 1D middle box]. To do this, we used the workflow shown in Figure 5A. We examined the probe count data: the number of probes giving a detectable signal across the 12 rat samples.

The Rat v1.5 miRNA codeset contains probes for 420 endogenous miRNAs (about half the number on the human codeset, in Figure 1C). The majority of probes gave a signal (count ≥ 2) in unprocessed raw count data prior to any background correction or normalisation. Only 14 probes failed to give a signal in any sample [Figure 5B black line]. Most of the remaining 406 probes could be detected in 8 of the 12 rat samples. The number of probes that could be detected in 9 or more rats dropped off, but 284 probes could be detected in all 12 rat samples [Figure 5B black line].

In common with any assay, a small percentage of counts for each target in a NanoString codeset will represent false positives due to non-specific binding. This can be problematic for the analysis of circulating miRNA due to the low expression levels. The background level is determined using 8 negative control probes present in the assay designed to recognise engineered RNA sequences that are not found in the biological sample [Figure 1C]. The background level set is calculated from the mean count of these negative controls. However, the level of stringency used in different published studies varies greatly, from simply using the mean value to using the mean and two standard deviations. The balance between false positive and false negative target occurrences will depend on this stringency.

We looked at the effect of applying a background subtraction on the number of probes that could be detected. When a background level set at the mean of the negative control probes [Supplementary Table 2] was applied to our dataset, the number of detected probes was substantially reduced [Figure 5B red line]. Only 229 probes could be detected in 1 or more rats, and this number dropped until only 63 probes gave a count of 2 or more for all 12 rat samples. Applying a more stringent background threshold further reduced the number of probes detected. With background at mean + 1 SD, only 36 probes were detected in all 12 rats [Figure 5B green line], with only 31 at mean + 2 SD [Figure 5B blue line].

Thus, applying a background subtraction substantially reduces the number of probes in the analysis, potentially leading to false negatives in the output.

### Effect of positive control normalisation on probe count

We next looked at the effect of normalising the data after background subtraction [Figure 1D and Figure 5A, C and D].

nSolver incorporates two normalisation steps to reduce sample variability. Positive control normalisation is used to control for any technical variation between samples. Samples are normalised using the readouts from 6 positive control probes spiked into the reaction mix at different concentrations [Figure 1C and Supplementary Table 3]. These count data are used to calculate a normalisation conversion factor, which we found varied from 0.78 to 1.19, and this is applied to the count data for all probes within a sample.

Applying this positive control normalisation to the dataset (background: mean + 2 SD) increased the number of probes above the threshold [Figure 5D green line]. All 420 probes were detectable in 5 or more rats, a number higher than for the raw count data (406). There was then a dramatic drop-off, with only 152 probes being detectable in 6 or more rats, followed by a more gradual decline in the number detected in more rats [Figure 5D green line].

### Identification of endogenous normalisers

The second normalisation step is based on endogenous expression and is known as content normalisation. NanoString recommends using one of two methods for content normalisation: these essentially involve normalising to a global average of all targets expressed above the threshold in all samples (referred to as the total RNA method) or alternatively identifying a small group of stably expressed targets within the cohort to use as normalisers (referred to as the NormFinder method)^[43,44]^.

To identify a set of probes to use as normalisers for the total RNA method, we applied a background subtraction of mean + 1 SD of negative control probes without positive control normalisation (the method recommended by Nanostring^[43]^). Thirty-six probes were expressed above this threshold in all 12 rats [Figure 5C and Supplementary Table 4]. Nanostring recommends removing any probe expressed below 50, but we did not do this because this would have reduced the cohort to only 20 probes. The most highly expressed normaliser, rno-miR-122, had an average expression of 1,786 counts, while the lowest, rno-miR-448 had an average expression of just 6 counts. We noted that this cohort included the red blood cell-specific miR-451, as well as miR-23a, but did not include either miR-191-5p or miR-103-3p [compare Supplementary Table 4  to Figure 2].

To identify a set of probes to use as normalisers for the Normfinder method, we used Normfinder to calculate the stability values of these 36 total RNA probes and selected the 5 most stable [Figure 5C and Supplementary Table 5].

### Effect of content normalisation on probe count

We calculated that the total RNA normalisation conversion factor ranged from 0.49 to 1.63 for each rat, with the control cohort exhibiting greater stability and the larger conversion factors being applied to the pregnant group. Removing miR-451 (possible contaminant from red blood cells) from the cohort altered the conversion factor by an average of 0.02 and a maximum of 0.05 (data not shown). We calculated that the Normfinder normalisation conversion factor ranged from 0.14 to 3.2, and again the larger conversion factors were applied to the pregnant group.

Applying content normalisation as well as positive control normalisation to the dataset [Figure 5A] had the effect of further increasing the number of probes above the threshold [Figure 5D]. Using total RNA normalisation resulted in all 420 probes being detected in 8 rats and 53 probes above the threshold in all 12 rats. Using Normfinder, all probes were detected in 10 or more rats and 86 in all [Figure 5D].

This analysis suggests that some probes below the level of detectability have been brought above the threshold by the normalisation factor conversion, potentially leading to false positives in the dataset.

### nSolver data processing

**Figure 6:**
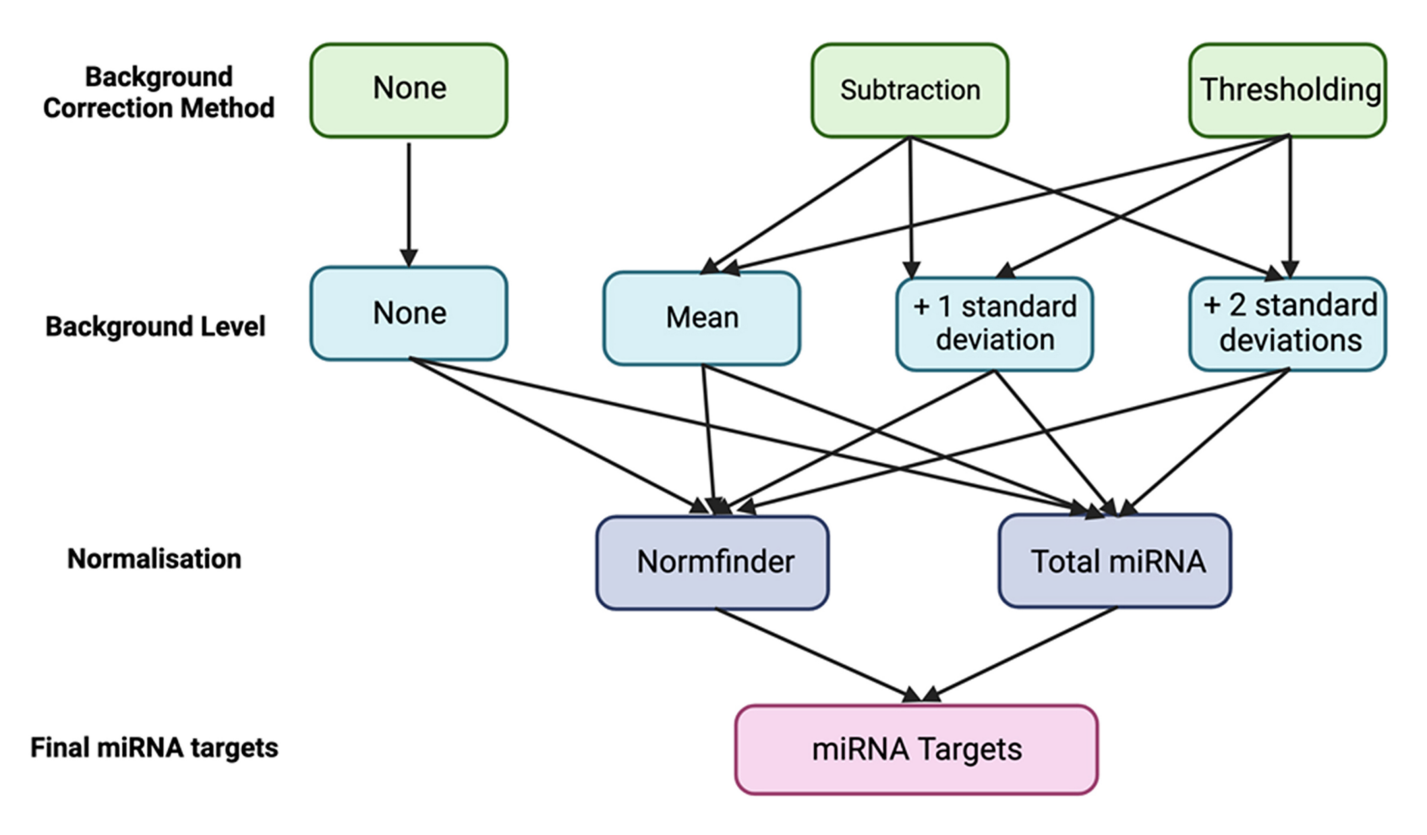
nSolver data processing workflows used in this analysis. Visual representation of the 14 different nSolver analysis workflows used in this study to process raw count data. These differed in the method of background correction (green), background level stringencies (light blue), and normalisation methods (purple), as indicated.

Having looked at the effect of background correction and normalisation on probe counts, we next developed workflows to process our raw count data using nSolver. We developed a total of 14 different analysis workflows [Figure 6 and Table 3]. Each workflow uses a different combination of user-defined variables within the nSolver. These are: the method of background correction (none, subtraction or thresholding) [Figure 6 green boxes], the stringency of the background level set [(mean of negative control probes, mean + 1 SD or mean + 2 SD) Figure 6 blue boxes], and the method of content normalisation (total RNA or Normfinder) [Figure 6 purple boxes].

**Table 3:** nSolver data processing workflows used in this analysis

**Method**	**1**	**2**	**3**	**4**	**5**	**6**	**7**	**8**	**9**	**10**	**11**	**12**	**13**	**14**
Background correction	N	N	S	S	S	S	S	S	T	T	T	T	T	T
Stringency	N	N	2	1	M	2	1	M	2	1	M	2	1	M
Normalisation	NF	TR	NF	NF	NF	TR	TR	TR	NF	NF	NF	TR	TR	TR

14 different nSolver analysis workflows were used in this study to process raw count data numbered 1-14. Background Correction, N: none; S: subtraction; T: thresholding. Stringency, N: not applicable; 2: mean + 2 standard deviations; 1: mean + 1 standard deviation; M: mean. Normalisation, NF: normfinder; TR: total RNA method.

The outputs from each of these 14 workflows were analysed using both multivariate (to visualise general trends in the data) and univariate methods (to identify differentially expressed miRNA) [Figure 1D middle box].

### Multivariate analysis

We first looked at general trends in the data by performing an unsupervised PCA. Large datasets are often difficult to interpret and PCA plots are a common tool used to reduce the size of such datasets, increasing interpretability while minimizing information loss without discarding any data points^[51]^. A PCA plot shows clusters of samples based on their similarity, projecting them into a 2D space to visualize any general trends in the data without knowing any a priori assumptions.

**Figure 7:**
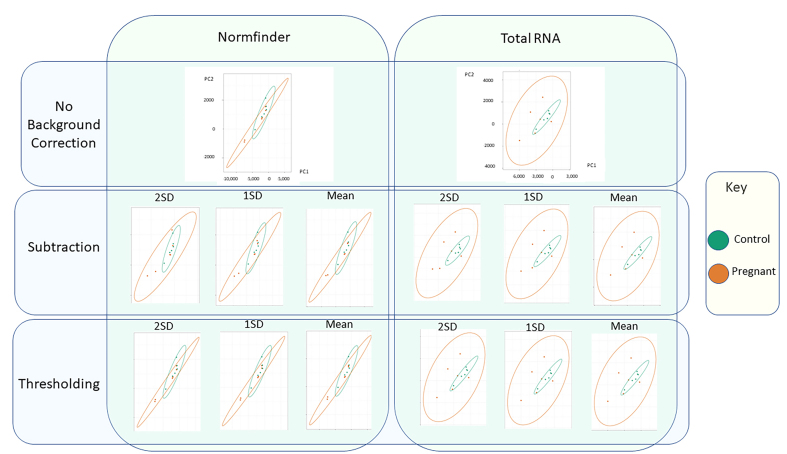
Multivariate analysis of nSolver processed data. Graphs show unsupervised PCA for each of the 14 nSolver workflows. A spot is shown for each rat and an ellipse shows the variation within each group. Green shows the control group and orange the pregnant. PCA: Principal component analysis.

The data revealed clustering and low separation of the two groups, indicating a lack of global differences [Figure 7]. In all cases, the variance of the pregnant group was larger than that of the control. Amongst the 14 analysis workflows, two clear patterns emerged corresponding to the Normfinder and total RNA content normalisation options. Normfinder processed data clustered more closely together and the two groups overlapped. In contrast, the total RNA processed data showed a broader spread and the control group was entirely contained within the larger pregnant group.

### Identification of differentially expressed miRNA by nSolver data analysis

We then used the output from all 14 workflows [Figure 6 and Table 3] in order to generate a list of putative differentially expressed miRNA [Figure 1D middle box]. The results were interesting. The 14 analysis workflows generated a total of 31 changed miRNAs [Table 4]. 10 miRNAs were enriched in pregnant rats, while 21 were higher in controls. No single miRNA was identified by all workflows and the most commonly identified target, miR-1224, was identified in only 7 of the 14 workflows. Furthermore, nearly half of the changes (14 miRNAs) were identified by only a single method. There was no clear pattern to the results: neither NormFinder (21 hits) nor the total miRNA (21 hits) method was clearly better than the other; the same was true for background thresholding (19 hits), subtraction (14 hits), or no background (8 hits). Furthermore, changing the background stringency level often resulted in a different set of targets being identified.

**Table 4:** Putative changed miRNA identified by NanoString global screening

			**Method**													
miRNA	Freq	Enriched in group	1	2	3	4	5	6	7	8	9	10	11	12	13	14
miR-1224	7	Pregnant			X	X		X	X		X			X	X	
miR-431	6	Control		X			X			X		X	X			X
miR-3563-5p	5	Control			X			X	X		X			X		
miR-3573-5p	4	Control				X			X			X			X	
miR-132	3	Control					X			X		X				
miR-872	3	Pregnant					X					X			X	
miR-125a-5p	3	Control					X					X	X			
miR-7d	3	Control					X				X	X				
miR-183	2	Pregnant	X	X												
miR-7b	2	Control				X	X									
miR-199a-3p	2	Control					X					X				
miR-99a	2	Control					X			X						
miR-450a	2	Pregnant						X	X							
miR-196c	2	Control											X			X
miR-3580-3p	2	Inconsistent											X		X	
miR-423	2	Pregnant											X			X
miR-26a	1	Pregnant		X												
miR-344a	1	Control		X												
miR-134	1	Pregnant		X												
miR-339-5p	1	Control		X												
miR-741-5p	1	Control		X												
miR-127	1	Control		X												
miR-19b	1	Pregnant						X								
miR-147	1	Control								X						
miR-93	1	Control									X					
miR-133a	1	Control										X				
miR-411	1	Control										X				
miR-3569	1	Control										X				
miR-301a	1	Control											X			
miR-350	1	Control											X			
miR-30a	1	Pregnant												X		

Each of the 14 nSolver analysis workflows is represented by a column and an X in that column signifies that the indicated miRNA was found to be significantly changed by that workflow. miRNAs are ranked by the frequency of occurrence across workflows. miRNA: MicroRNA; NF: normfinder method; TR: total RNA method; 1sd: mean + 1 standard deviation; 2sd: mean + 2 standard deviations.

### Post-hoc analysis of haemoglobin and platelet contamination

To eliminate any possibility that our results could be biased by either red blood cell- or platelet-derived miRNA, we re-examined our data [Figure 1D middle].

Pre-hybridisation quality control demonstrated all of our samples are well below the haemolysis threshold determined by Blondal [Figure 4]. However, we noted that this analysis is dependent on the use of miR-23a as a sample normaliser. This miRNA exhibited the highest coefficient of variance of those tested [Figure 3C and F], and is not one of the most stable miRNA identified by Normfinder [Supplementary Table 5]. This suggests that miR-23a may not be a suitable endogenous normaliser. Therefore, we examined the expression data for miR-451a using our 14 nSolver workflows incorporating the total RNA and Normfinder endogenous normalisers. None of these workflows revealed a significant change in miR-451a [Table 3], indicating haemolysis was not a problem and providing confidence in the pre-hybridisation quality control data.

Platelets are a second potential source of contamination in blood samples, particularly in serum, as platelets are known to release miRNA within extracellular vesicles during clotting. While numerous studies support the hypothesis that miR-451a is strongly and specifically expressed in red blood cells^[52]^, there is less consensus on specific markers of platelet contamination. Sunderland *et al.*^[53]^ compared the data reported in 8 studies to establish the most consistently reported miRNA highly expressed in platelets. They report considerable variation between individual studies but report the top 5 as miR-126-3p, miR-191-5p, miR-16-5p, miR-24-3p, and miR-223-3p. Teruel-Montoya^[54]^ compared miRNA expression levels between platelets and blood cell types, revealing that not all of those identified by Sunderland are specific to platelets. For example, miR-223-3p was shown to be expressed in granulocytes. Taken together, these studies point to miR-126-3p being a strongly expressed largely-specific marker of platelets (although it has also been reported to be expressed in endothelium lining blood vessels^[55]^).

We, therefore, examined the expression of miR-126 within our nCounter data. miR-126 was not found to be significantly different between cohorts by any of the 14 nSolver workflows [Table 3], indicating no contamination from platelets.

### RT-qPCR validation of candidate changes

Technical validation of a subset of candidate miRNAs identified in the above nSolver analysis was performed using RT-qPCR [Figure 1D lower box]. The miRNA chosen for validation were the 14 most commonly repeating candidates amongst the 14 analysis workflows. This included all 8 miRNA candidates identified by 3 or more workflows, together with a selection of those identified by 2 workflows (miR-183, miR-7b, miR-450a, miR-196c, miR-423) and one of the miRNAs identified in only a single workflow (miR-133a), chosen because it appears to be highly expressed. The probe for miR-3563-5p did not produce a detectable signal, and therefore, this miRNA was excluded from the analysis.

Although RT-qPCR is commonly used to validate genomics assay results, it should be noted that the results obtained from such an assay are themselves influenced by user-defined variables in much the same way as the nSolver analysis described above.

We used a variant of the standard 2^-ΔΔCT^ method^[56]^. In this method, a threshold is defined during the exponential phase of amplification and the point at which the amplification curve for a given probe crosses this threshold is called the quantification cycle (Cq, formerly known as cycle threshold, Ct). The Cq value of each test miRNA is compared to that of a reference miRNA to control for differences in the amount of starting material. As noted above, one problem encountered when analysing plasma miRNA is the lack of established endogenous normalisers expressed at a stable level. The choice of this reference gene is, therefore, one user-defined variable that may influence the output. We chose as our “reference gene” the mean of two miRNAs identified to be the most stable within the nSolver raw dataset by NormFinder analysis (the probes for miR-20a/miR-20b-5p and miR-27b) [Supplementary Table 5], a method that has been used previously in such assays^[17]^.

The miRcury RT-qPCR kit includes a synthetic spike-in (UniSP6) to control for potential technical variation due to differences in reverse transcription efficiency. We looked at the effect of subtracting the UniSP6 Cq value from all probe Cq values prior to subtracting the reference gene Cq from the test probe Cq, but found that the resulting ΔCq values were identical to those obtained by simply subtracting the reference Cq from the test probe Cq (data not shown).

There is disagreement in the literature as to whether statistical analysis should be performed on the ΔCq values (which are base 2 logarithmic numbers) or on the log-transformed 2^-ΔCq^ numbers (which are linear)^[57,58]^. We observed that different results could be obtained from these two options; therefore, this represents another user-defined variable that may influence the output.

Another variable is the choice of parametric *vs.* non-parametric statistics. An assumption of a parametric test such as a *t*-test is that the data are normally distributed, and thus, any probes for which data points are not normally distributed must be assessed using the non-parametric Mann–Whitney or Wilcoxon test. However, these tests are not directly comparable, and within a dataset, there may be a mix of parametric and non-parametric distributions for individual probes. We used the Shapiro Wilk test to determine whether the data for each probe/group were normally distributed and then selected as appropriate a *t*-test or a Mann–Whitney test.

Finally, another user-defined variable is the level of stringency, determined here by the choice of two-tailed or one-tailed statistics.

**Figure 8:**
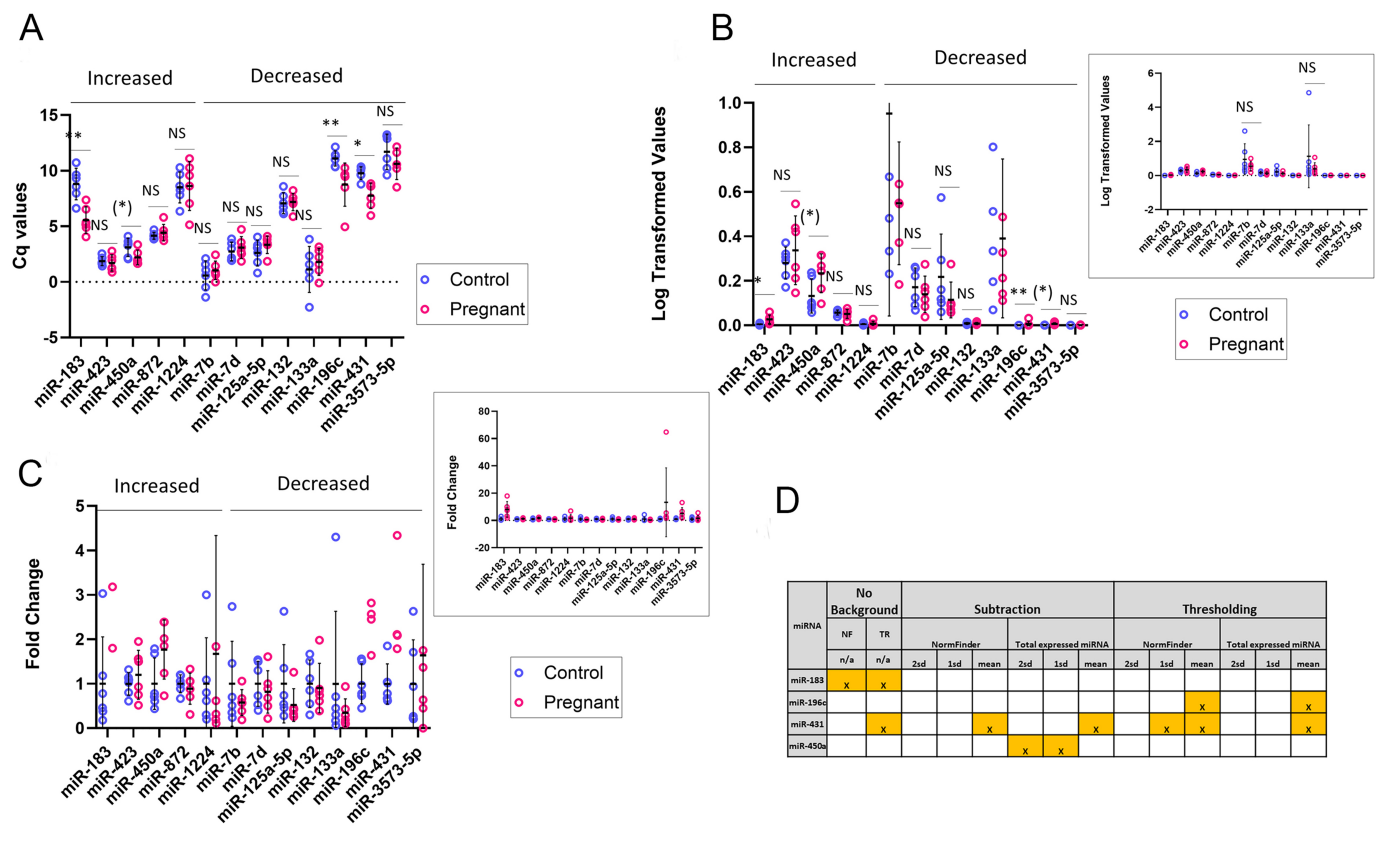
Validation of miRNA expression changes. miRNA: MicroRNA; Cq: quantification cycle; A-C: target validation by miRcury RT-qPCR. Three technical replicates were performed for each of 6 biological samples per group. The mean value of each biological sample is plotted as a circle (blue: control; red: pregnant). Bars show the mean and standard deviation for each group. miRNAs are grouped as Increased or Decreased in reference to the change observed in pregnancy relative to controls in the nCounter assay. ^*^P < 0.05 in 2-tailed test; ^**^P < 0.005 in 2-tailed test; (^*^) *P* < 0.05 in 1-tailed test; NS: not significant; A: ΔCq values are plotted; B: 2^-ΔCq^ values are plotted. Inset shows the same data plotted on a different y-axis scale; C: Fold change values are plotted. Mean expression in control groups was set to 1 for each probe. Inset shows the same data plotted on a different y-axis scale; D: Table showing the nSolver analysis results for the 4 validated changes. Workflows correctly identifying each miRNA are highlighted in yellow and marked with an X.

We began by performing the analysis using the ΔCq values [Figure 8A]. The Shapiro-Wilk test of normality indicated that ΔCq values were normally distributed in control rats for all probes. In the case of pregnant rats, all except probe miR-196c were normally distributed. Therefore, the data for miR-196c were tested using a Mann-Whitney test and the remaining probes were tested using a *t*-test. The results revealed 4 significant changes with a *P*-value below the threshold of 0.05 when a one-tailed test was performed; this was reduced to 3 significant changes if a two-tailed test was applied [Table 5 and Figure 8A].

**Table 5:** Four miRNA changes were validated by RTqPCR

**Probe**	**Fold change**	**ΔCq values**		**2^-ΔCq^ values**	
		**Two-tailed**	**One-tailed**	**Two-tailed**	**One-tailed**
miR-183	8.08 +/- 5.75	*t*-test, *P* = 0.0017	*t*-test, *P* = 0.0009	*t*-test, *P* = 0.0290	*t*-test, *P* = 0.0145
miR-196c	13.30 +/- 25.25	MW, *P* = 0.0020	MW, *P* = 0.0010	MW, *P* = 0.0020	MW, *P* = 0.0010
miR-431	5.01 +/- 4.38	*t*-test, *P* = 0.0054	*t*-test, *P* = 0.0027	*t*-test, *P* = 0.0749	*t*-test, *P* = 0.0375
miR-450a	1.77 +/- 0.66	*t*-test, *P* = 0.0602	*t*-test, *P* = 0.0307	*t*-test, *P* = 0.0578	*t*-test, *P* = 0.0289

The table shows a summary of statistical analysis of RTqPCR data. miRNA: MicroRNA; Cq: quantification cycle; MW: Mann-Whitney test.

We then analysed the log-transformed 2^-ΔCq^ values. When plotted on a linear rather than a log scale [Figure 8B], outliers are further from the mean value, and as a result, fewer probes showed a normal Cq distribution. Five probes were analysed using a Mann-Whitney test and the remaining 8 using a *t*-test. *P*-values obtained for all probes using a *t*-test were higher than those obtained when analysing ΔCq values, while *P*-values for the Mann-Whitney test, which ranks data points rather than looking at the actual values, were unchanged [Table 5]. As a result, only 2 probes were found to have *P* -values below the 0.05 threshold when analysed with a two-tailed test, but the same 4 probes were found to be significant when using one-tailed statistics [Table 5].

Finally, we divided the 2^-ΔCq^ values of the pregnant group by that of the control group to obtain fold change values [Table 5 and Figure 8C]. Of the 13 tested miRNAs, 9 (miR-183, miR-1224, miR-450a, miR-423, miR-7b, miR-7d, miR-133a, miR-125a-5p, miR-132) showed a change in the same direction as that detected by the NanoString assay [Figure 8C]. Surprisingly, 4 miRNAs exhibited the opposite effect (miR-196c, miR-431, miR-872, miR-3573-5p; Fig 8c), and this includes 2 of the validated changes (miR-196c, miR-431). It is also noteworthy that one of the validated changes, miR-450a, is one of the cohort of 36 miRNAs used to normalise the nSolver data by the total RNA method [Supplementary Table 4].

Thus, the technical validation revealed that only a minority of the putative differentially expressed miRNAs identified by nSolver analysis were confirmed.

### *In silico* recalculation analysis of nSolver workflow data processing

The validation analysis presented above did not immediately suggest an optimal nSolver analysis workflow. None of the 14 workflows alone was sufficient to identify all 4 validated changes [Figure 8D]. miR-431 was identified in a total of 6 workflows, ranking as the second most significantly changed miRNA in our analysis [Table 4]. However, only three of these 6 workflows successfully identified another of our validated changes. miR-183 was also identified by a second workflow unique to this changed miRNA, while neither of the workflows that identified miR-450a picked up any of the other validated changes [Figure 8D]

To further understand nSolver data processing steps, a mathematical *in silico* recalculation was performed [Figure 1D lower box]. By following the information provided in NanoString tech notes, we manually recalculated the effect of each step within the workflows for the 4 validated miRNA changes (miR-450a, miR-183, miR-196c and miR-431) and compared these to the results given by nSolver in order to gain insights into this “black box” process.

**Figure 9:**
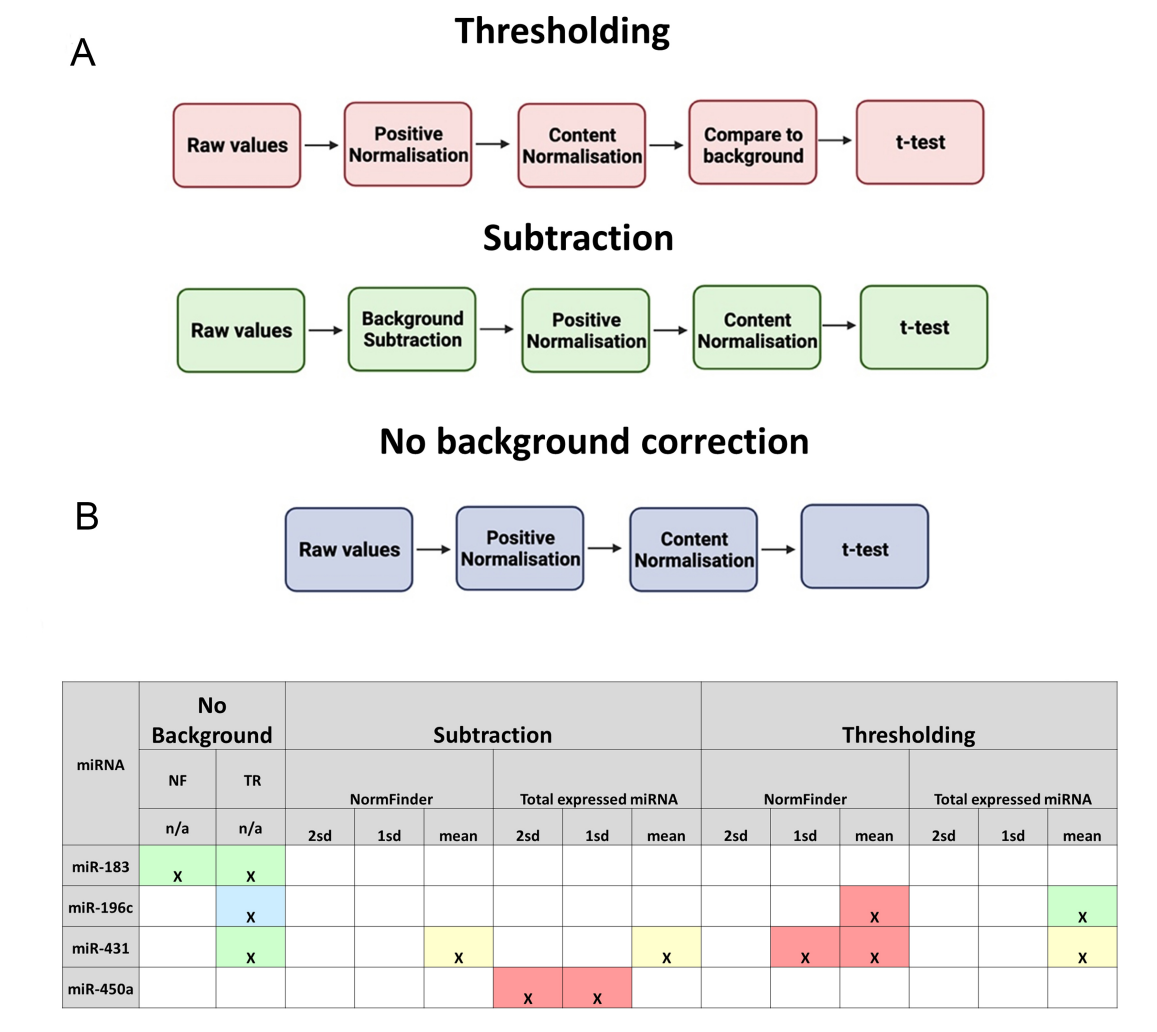
*In silico* recalculation of nSolver data processing. NF: Normfinder; TR: total RNA method; A: flowcharts to illustrate differences in the order of data processing steps within the three workflow subtypes; B: results of recalculations for the 4 validated miRNA changes. × indicates a nSolver automated workflow that identified a significant change in the given mRNA. Colour coding indicates the results of recalculation. green: recalculation agrees with nSolver; red: recalculation does not agree (nSolver positive, recalculation negative); blue: recalculation does not agree (nSolver negative, recalculation positive); yellow: recalculation *t*-test agrees but errors found in calculation.

By obtaining nSolver outputs for intermediate steps in the analysis, we were able to determine the correct order of steps performed by nSolver [Figure 9A], enabling us to develop a recalculation formula for each. This revealed that in thresholding workflows, normalisation is performed before background correction, but in subtraction workflows, these steps are reversed.

We first looked at thresholding workflows [Figure 9A top row]. Supplementary Tables 6 and 7 show our recalculations of the normalisation factors for the Normfinder and total RNA methods, respectively. Supplementary Tables 8 and 9 show the effect on the raw count data of each step in the thresholding workflows for the Normfinder and total RNA methods, respectively. The first step in the thresholding analysis workflows was the positive control normalisation of the raw counts, followed by content normalisation (either using NormFinder or total miRNA normalisation targets). Once these values were obtained, they were compared to the background at each of the 3 thresholds used in our workflows (mean of negative controls, mean + 1 SD or mean + 2 SD) and if the value obtained fell below the background value for that lane, the value was replaced by the background value as a threshold. Finally, Welch’s *t*-test was used to compare the two groups.

Most of the recalculated values match those reported by nSolver [Supplementary Tables 8 and 9], which confirms the accuracy of the identified formula. However, we noted that occasionally, there was a difference between the recalculated and nSolver values [Supplementary Tables 8 and 9 values shown in red]. In the case of the thresholding plus total expressed RNA workflows, these errors did not affect the final *t*-test result: our results agreed with nSolver that miR-431 and miR-196c were significantly changed using a background threshold set to the mean, while other thresholds, as well as all results for miR-450a and miR-181, were not significant [Supplementary Table 9 and Figure 9B]. However, in the case of the thresholding plus NormFinder workflows, these count changes did affect the results. For miR431, nSolver deemed the workflow statistically significant when using a threshold of either mean or mean + 1 SD, but the recalculation did not, while the same was true for miR-196c when using a threshold of the mean [Supplementary Table 8 red highlighting and Figure 9B]. This was due to a large change in the final counts for two of the pregnant rat samples. The recalculated results for miR450a and miR183 agreed with that of nSolver.

We next looked at the subtraction workflows [Figure 9A middle row]. Unlike the thresholding workflows, background correction is the first step in the subtraction analysis workflows [Figure 9A]. Supplementary Tables 10 and 11 show the effect on the raw count data of each step in the subtraction workflows for the Normfinder and total RNA methods, respectively. The first step in the subtraction analysis workflows was to subtract the background levels for each lane from the raw counts, followed by positive control normalisation and then content normalisation (once again using either NormFinder or total miRNA normalisation). Finally, Welch’s *t*-test was used to compare the two groups.

In these subtraction workflows, we observed many differences in processed count values between the recalculated figures and nSolver values [Supplementary Tables 10 and 11 values shown in red]. This did not affect the final outcome of the subtraction plus NormFinder workflows [Supplementary Table 10]: our results agreed with nSolver that the only significant change was miR-431 when using a background threshold set to the mean. However, in the subtraction plus total miRNA workflows [Supplementary Table 11], there were discrepancies: while the significant result for mir-431 using mean background was again replicated (green highlighting), the result for miR450a was not (red highlighting).

Finally, we looked at the background-free adjustment workflows [Figure 9A bottom row]. In these workflows, the background levels are ignored and the raw values are directly subjected to positive normalisation followed by content normalisation (once again, using either NormFinder or total miRNA normalisation) [Figure 9A]. For these workflows, we found no errors in the calculations for miR-183, miR-450a, and miR-431 and our results matched that of nSolver [Supplementary Tables 12 and 13]. However, we did find errors in the calculation for miR-196c, and this resulted in our recalculation finding a significant change for this miRNA that was not picked up by nSolver [Supplementary Table 13 blue highlighting and Figure 9B].

## DISCUSSION

In this study, we report the utility of the Nanostring nCounter assay for detecting differences in circulating miRNA in maternal blood between two conditions. We examined the effect of user-defined variables within the nSolver data analysis workflow such as background correction, background threshold, and content normalisation [Figures 5 and 6] upon the output. We report that these variables have a substantial influence on the output and that each combination produces a different set of reported changes [Table 3]. Our results lead us to suggest that investigators in the future should not rely on a single analysis method to identify changes.

The four validated differentially expressed miRNAs [Figure 8 and Table 5] have known roles in pregnancy, giving us confidence in the final result. miR-183, miR196, and miR-450a are expressed within extracellular vesicles released by the placental syncytiotrophoblast and can be detected in the blood of pregnant mice (miR-183, miR-450a)^[59]^ or human patients^[60]^. miR-183 was found to be one of the most enriched miRNAs in such vesicles. miR-183 has also been reported in blood-derived extracellular vesicles from pregnant cows^[61]^. miR-183 has been shown to regulate uterine receptivity and enhance embryo implantation in both an *in vitro* Ishikawa endometrium cell model and in an *in vivo* mouse pregnancy model^[62]^, while the human homologue of miR-196c has been shown to influence macrophages^[60]^. These studies strongly support our finding of increased expression of these miRNAs in maternal blood during pregnancy. miR-431 has been found to be overexpressed within the placenta of preeclampsia patients^[63,64]^, and it has been suggested to inhibit the migration and invasion of trophoblastic cells, which may give rise to preeclampsia^[64]^. However, we are not aware of studies demonstrating its presence in maternal blood and it is noteworthy that our results for this miRNA were ambiguous, with nCounter suggesting decreased expression in pregnancy, which is the opposite of the RT-qPCR result.

Our analysis demonstrated that user-defined variables significantly impact the output produced by nSolver [Table 3 and Figure 8C]. No single analysis workflow was able to correctly identify the 4 validated miRNA changes and even the most frequently seen change (miR-1224) occurred in only half of the workflows. Furthermore, the validated miRNAs were not those most frequently occurring in the various workflows.

Most published Nanostring studies use only a single analysis method, and often, this choice is not explained. NanoString recommends Thresholding as the method of choice for most analytes and acknowledges that Subtraction can lead to false positives^[42]^. It is, therefore, surprising that a number of published studies have utilised Subtraction as the sole method in their analysis^[33,37,65,66]^. Our data indicate that Thresholding picked up only one of the four validated changes [Figure 8C], suggesting that this method leads to false negatives. Subtraction plus total RNA normalisation was able to pick up 2 of the 4 validated targets, albeit at different levels of background. No Background plus total RNA normalisation was able to pick up 3 of the 4 validated changes, while the fourth validated target, miR-450a, was only just above the *P* = 0.05 threshold. This is consistent with the fact that the expression of miRNA is generally at a very low level, and therefore, any background correction will produce false negatives. However, if no background threshold is applied, the system is likely to pick up false positives, something that we did not test in our recalculation. Thus, our data suggest that investigators should analyse their data using a range of workflows including Thresholding and Subtraction, as well as No Background, in order to pick up all potential changes.

*In silico* recalculation revealed errors in nSolver data processing [Figure 9B], which compound the inherent problems associated with background correction. Why was there a discrepancy in many processed counts between those reported by nSolver and those in our recalculation? We can only speculate on the reasons behind this. It is surprising that errors occurred in some lanes and not others, given that the same formula must have been applied to each. It is possible that rounding errors occur in these workflows that accumulate over the different steps, which leads to false values at the end. However, the analysis suggests this is not always the case. In some cases, nSolver appears to have simply made a mistake. In some of the thresholding workflows, nSolver appears to have replaced the correct output with the corresponding background value even in cases where the experimental value was higher than background, and thus, there was no need to threshold to background. It is noteworthy that errors were made only in the Thresholding and Subtraction workflows, and no errors were made in the No Background correction workflows [Figure 9B]. This suggests that the more complex the workflow, the higher chance of an error by nSolver.

A surprising number of published studies have reported unvalidated nCounter data^[14,31,32,34]^. Our results, in common with others, suggest that technical validation is a critical step. For example, in a study of pre-term birth, Kim *et al.* technically validated 4 of 7 miRNA changes, of which only 2 were subsequently validated in an independent biological cohort^[17]^. Filardi *et al.*, in a study of gestational diabetes, found that only 2 of 12 changes were validated in an independent cohort^[33]^. In our study, only 4 of 13 changes were technically validated [Figure 8].

It is interesting to note that variability in output associated with differing analysis methods is not limited to the nCounter assay, but is perhaps a common feature of many genomic assays. We show here that user-defined variables also have an influence on the reported results of RTqPCR assays, while others have demonstrated that the results of RNAseq are similarly influenced by user-defined variables^[67]^.

One limitation of our study is that we were not able to directly compare the output of the nCounter assay to an alternative, such as an RNAseq assay. It would be informative to perform such a test in the future. The cost of the two assays is similar. A clear advantage of RNAseq is the ability to perform an unbiased screen in order to pick up any expressed miRNA, in contrast to the subset of probes included in the nCounter assay. This must be weighed against the simpler data processing required for the nCounter assay. Although we looked at two different normalisation methods for the nCounter assay, we did not do the same for the RTqPCR assay. It would be valuable in the future to investigate the effect of this choice on the output. We were also limited by small group size, and it is likely that a better powered study would identify a greater number of significant changes. Finally, we assayed the total circulating RNA in plasma, which includes both protein-bound and vesicle-bound extracellular RNA. Our assay did not distinguish between these two sources, and in future work, it would be useful to do so.

In conclusion, we have demonstrated that NanoString nCounter profiling can be used successfully as a global screening tool to identify changes in circulating miRNA and is, therefore, a potentially useful tool for biomarker identification. However, it is critical to understand that user-defined variables within the nSolver workflow greatly affect the final output. Clearly, standardised methods are needed in the field. We propose that researchers use multiple analysis workflows to identify changes and validate these changes with RT-qPCR.

## DECLARATIONS

### Availability of data and materials

Raw data have been uploaded to the Gene Expression Omnibus (GEO accession number: GSE267016).

### Copyright

© The Author(s) 2024.
