# Supplementary material for: Assessment of NanoString technology as a tool for profiling circulating miRNA in maternal blood during pregnancy
Source: Extracell Vesicles Circ Nucl Acids. 2024 Sep 5;5(3):471–96. doi: 10.20517/evcna.2024.38 (PMC11648433; doi:10.20517/evcna.2024.38)
Supplement: Supplementary file 1 [file evcna-5-3-471-SupplementaryMaterials.pdf]

Supplementary Materials

Assessment of NanoString technology as a tool for profiling circulating miRNA in maternal blood during pregnancy

Petra Adamova[1,2], Andrew K. Powell[1,2], Iain M. Dykes[1,2]

[1]Department of Pharmacy and Biomolecular Sciences, Liverpool John Moores University, Liverpool L3 3AF. UK.
[2]Liverpool Centre for Cardiovascular Science, Institute for Health Research, Liverpool John Moores University, Liverpool L3 3AF. UK.

Correspondence to: Dr. Iain M. Dykes, Department of Pharmacy and Biomolecular Sciences, Liverpool John Moores University, Byrom St, Liverpool L3 3AF. UK.
E-mail: i.m.dykes@ljmu.ac.uk

Supplementary Table 1. Post-hybridisation quality control metrics

Group	Sample	Imaging QC	Binding density QC	Positive Control Linearity QC	Limit of detection QC of RNA	Ligation QC	FOV count	FOV counted
Control	R5	0.99	0.16	0.98	29.03	29.03	555	551
	R8	0.99	0.14	0.99	27.43	27.43	555	550
	R20	0.99	0.15	0.99	23.88	23.88	555	548
	R22	0.99	0.14	0.98	25.03	25.03	555	547
	R23	0.97	0.13	0.99	22.2	22.2	555	538
	R24	0.98	0.15	0.99	22.83	22.83	555	545
Pregnant	R9	0.99	0.15	0.99	38.69	38.69	555	549
	R11	0.99	0.16	0.99	27.32	27.32	555	550
	R12	0.99	0.15	0.99	27.07	27.07	555	550
	R14	0.99	0.14	0.99	24.41	24.41	555	551
	R18	0.99	0.17	0.99	26.48	26.48	555	547
	R19	0.99	0.16	0.99	30.58	30.58	555	547

Supplementary Table 2. Negative control probe data and background level calculation

	Control						Pregnant					
	R5	R8	R20	R22	R23	R24	R9	R11	R12	R14	R18	R19
NEG_A	19	11	13	10	16	16	12	18	18	10	21	16
NEG_B	18	10	10	3	11	11	6	7	10	8	13	16
NEG_C	8	11	10	16	3	9	9	11	14	5	16	11
NEG_D	8	10	13	11	7	8	13	12	13	6	10	7
NEG_E	11	19	13	17	10	17	20	15	13	18	16	16
NEG_F	21	19	21	11	12	12	31	20	19	16	18	25

**NEG_G**	10	6	12	4	3	7	9	12	5	5	10	10
**NEG_H**	10	4	15	9	6	14	6	6	7	7	11	13
**Mean**	13.13	11.25	13.38	10.13	8.50	11.75	13.25	12.63	12.38	9.38	14.38	14.25
**SD**	5.30	5.39	3.50	4.97	4.57	3.69	8.48	4.90	4.90	5.01	4.03	5.44
**Mean + 1SD**	18.43	16.64	16.88	15.09	13.07	15.44	21.73	17.52	17.27	14.39	18.41	19.69
**Mean + 2SD**	23.73	22.03	20.38	20.06	17.63	19.14	30.21	22.42	22.17	19.40	22.44	25.14

The table shows the raw count data for the 8 synthetic negative control probes included in the nCounter probeset (Neg_A to Neg_H). The level of background for each test sample is calculated from the mean of these values. In some calculations, a higher level of stringency is applied by adding one or two standard deviations to the mean.

Supplementary Table 3. Positive control probe data and calculation of normalisation factor

	**Control**						**Pregnant**					
	**R5**	**R8**	**R20**	**R22**	**R23**	**R24**	**R9**	**R11**	**R12**	**R14**	**R18**	**R19**
**POS_A**	47617	43441	47800	42067	34455	46668	50447	53906	46764	39390	44554	44669
**POS_B**	16240	14336	15724	14036	11697	14910	16385	17619	15345	13360	14757	14650
**POS_C**	4399	3890	4193	3840	3248	4192	4439	4794	4230	3623	3932	3937
**POS_D**	873	798	932	842	736	891	909	1078	864	757	846	909
**POS_E**	117	122	128	106	106	145	127	148	123	107	118	125
**POS_F**	115	104	130	104	72	100	117	123	91	93	133	129
**GeoMean**	1849	1704	1912	1661	1394	1831	1916	2114	1756	1559	1803	1832
**Arithmetic mean of GeoMeans**	1778											
**Positive Control Normalisation**	1.04	0.96	1.08	0.93	0.78	1.03	1.08	1.19	0.99	0.88	1.01	1.03

Factor													

The table shows the raw count data for the 6 synthetic positive control probes included in the nCounter probeset (Pos_A to Pos_H). nSolver calculates the geometric mean (GeoMean) for each lane, followed by the arithmetic mean of all samples. The positive control normalisation factor is then calculated for each lane by dividing the lane-specific GeoMean by the arithmetic mean.

Supplementary Table 4. Set of 36 miRNAs used for nCounter sample normalisation with the Total miRNA method.

Probe Name	Accession #	% Samples above Threshold	Count after subtraction			%CV
			Avg	Min	Max	
rno-miR-122	MIMAT0000827	100	1786	49	5697	111
rno-miR-22	MIMAT0000791	100	679	58	2167	96
rno-miR-451	MIMAT0001633	100	600	30	2511	115
rno-miR-150	MIMAT0000853	100	551	30	1858	113
**rno-miR-20a+rno-miR-20b-5p**	MIMAT0000602	100	292	20	811	83
rno-miR-27b	MIMAT0000798	100	262	9	1157	127
rno-miR-23a	MIMAT0000792	100	203	14	985	133
rno-let-7b	MIMAT0000775	100	194	12	633	94
rno-miR-25	MIMAT0000795	100	192	3	665	107
rno-miR-27a	MIMAT0000799	100	170	17	635	107
rno-miR-449c-3p	MIMAT0017804	100	132	107	157	11
rno-miR-130a	MIMAT0000836	100	129	10	486	98
rno-miR-3594-5p	MIMAT0017898	100	92	71	119	13
rno-miR-3593-5p	MIMAT0017896	100	79	57	109	19
rno-miR-450a	MIMAT0001547	100	74	30	243	93
rno-miR-3590-5p	MIMAT0017889	100	71	51	117	29
rno-miR-421	MIMAT0001320	100	69	41	95	21

rno-miR-3596c	MIMAT0017877	100	62	47	78	14
rno-miR-466d	MIMAT0017824	100	55	44	76	17
rno-miR-455	MIMAT0005316	100	50	33	68	20
rno-miR-501	MIMAT0003116	100	49	32	71	24
rno-miR-466b	MIMAT0005278	100	38	25	57	22
rno-miR-3589	MIMAT0017888	100	34	25	46	18
rno-miR-539	MIMAT0003176	100	31	20	54	40
rno-miR-3595	MIMAT0017903	100	30	23	34	11
rno-miR-3578	MIMAT0017866	100	26	15	36	30
rno-miR-465	MIMAT0012850	100	24	13	41	29
rno-miR-3571	MIMAT0017851	100	21	15	27	21
rno-miR-3585-3p	MIMAT0017879	100	20	13	27	23
rno-miR-3592	MIMAT0017895	100	20	10	26	25
rno-miR-3581	MIMAT0017870	100	17	10	35	38
rno-miR-3590-3p	MIMAT0017890	100	16	7	31	41
rno-miR-3579	MIMAT0017867	100	13	9	20	23
rno-miR-543	MIMAT0004787	100	9	1	23	64
rno-miR-376b-5p	MIMAT0003195	100	9	1	18	75
rno-miR-448	MIMAT0001534	100	6	1	17	82

These were identified by applying a background subtraction of mean + 1SD of negative control probes without positive control normalisation.

Supplementary Table 5. The most stably expressed miRNAs identified by NormFinder software

miRNA name	NormFinder Stability Value	Count after subtraction		
		Avg	Min	Max
miR-20a+miR-20b-5p	0.076	292	20	811
miR-27b	0.121	262	9	1157

miR-27a	0.146	170	17	635
miR-450a	0.146	74	30	243
miR-22	0.151	679	58	2167

Lower values indicate the most stable expression. These 5 miRNAs were used for normalisation of samples in nSolver using the Normfinder method. In addition, the two most stable miRNA were used as loading controls to normalise RT-qPCR data.

Supplementary Table 6. *In silico* recalculation: NormFinder content normalisation calculation

	Control						Pregnant					
	**R5**	**R8**	**R20**	**R22**	**R23**	**R24**	**R9**	**R11**	**R12**	**R14**	**R18**	**R19**
**rno-miR-20a+ rno-miR-20b-5p**	204	169	309	366	179	192	105	38	237	324	753	831
**rno-miR-27a**	120	139	140	167	140	58	43	35	92	209	444	655
**rno-miR-27b**	197	169	166	179	146	99	65	27	201	198	727	1177
**rno-miR-450a**	75	54	64	62	48	60	61	48	58	89	208	263
**rno-miR-22**	493	572	437	353	332	132	308	76	1029	700	1736	2187
**Positive NF**	1.04	0.96	1.08	0.93	0.78	1.03	1.08	1.19	0.99	0.88	1.01	1.03
**rno-miR-20a+ rno-miR-20b-5p**	196	176	287	392	228	186	97	32	240	369	743	807
**rno-miR-27a**	115	145	130	179	178	56	40	29	93	238	437	636
**rno-miR-27b**	189	176	154	192	186	96	60	23	203	226	717	1142
**rno-miR-450a**	72	56	60	66	61	58	57	40	59	101	205	255
**rno-miR-22**	474	597	406	378	423	128	286	64	1042	798	1712	2123
**GeoMean**	171	172	169	202	181	94	82	35	194	276	606	795
**Arithmetic mean of**	248											

**GeoMeans**												
**NormFinder Normalisation Factor**	0.69	0.69	0.68	0.81	0.73	0.38	0.33	0.14	0.78	1.11	2.44	3.20

The raw count values of the 5 miRNAs identified by NormFinder were first divided by the positive control normalisation factor [Supplementary Table 3], the geometric mean (GeoMean) was calculated, and the arithmetic mean of these GeoMeans was taken. The NormFinder normalisation factor was then calculated by dividing the corresponding GeoMean by the arithmetic mean.

Supplementary Table 7. *In silico* recalculation: Total RNA content normalisation calculation

	Control						Pregnant					
	**R5**	**R8**	**R20**	**R22**	**R23**	**R24**	**R9**	**R11**	**R12**	**R14**	**R18**	**R19**
**rno-let-7b**	410	225	239	120	99	115	107	30	86	80	369	653
**rno-miR-122**	682	2632	1276	190	129	115	2599	67	5714	1760	1071	5397
**rno-miR-130a**	168	197	132	118	67	52	32	32	108	178	161	506
**rno-miR-150**	151	178	348	571	591	290	112	48	267	567	1815	1878
**rno-miR-20a+ rno-miR-20b-5p**	204	169	309	366	179	192	105	38	237	324	753	831
**rno-miR-22**	493	572	437	353	332	132	308	76	1029	700	1736	2187
**rno-miR-23a**	197	145	110	152	132	89	36	32	94	184	461	1005
**rno-miR-25**	134	98	133	204	109	173	69	21	113	187	683	589
**rno-miR-27a**	120	139	140	167	140	58	43	35	92	209	444	655
**rno-miR-27b**	197	169	166	179	146	99	65	27	201	198	727	1177
**rno-miR-3571**	45	35	43	32	31	30	47	44	43	35	38	37

**rno-miR-3578**	53	37	34	43	31	44	51	51	32	50	52	37
**rno-miR-3579**	34	31	30	27	33	27	31	29	29	27	29	30
**rno-miR-3581**	36	35	34	25	31	35	34	53	30	26	40	35
**rno-miR-3585-3p**	35	32	42	35	29	28	44	42	36	33	42	47
**rno-miR-3589**	58	49	42	51	43	61	58	55	49	43	44	58
**rno-miR-3590-3p**	49	33	36	25	22	27	37	37	35	21	43	37
**rno-miR-3590-5p**	91	83	96	73	64	120	82	78	82	66	135	83
**rno-miR-3592**	44	37	43	33	23	33	42	42	37	32	41	33
**rno-miR-3593-5p**	114	98	95	86	88	108	79	127	92	74	106	88
**rno-miR-3594-5p**	137	98	119	104	99	106	121	108	105	85	121	110
**rno-miR-3595**	52	46	45	49	46	48	49	46	48	42	41	52
**rno-miR-3596c**	84	71	81	68	73	86	83	86	64	73	87	98
**rno-miR-376b-5p**	36	24	29	21	15	32	23	35	23	17	20	32
**rno-miR-421**	113	79	102	86	54	75	89	89	70	93	95	87
**rno-miR-448**	32	19	21	18	15	21	23	35	26	21	20	31
**rno-miR-449c-3p**	161	146	146	148	120	146	179	147	147	121	161	162
**rno-miR-450a**	75	54	64	62	48	60	61	48	58	89	208	263
**rno-miR-451**	408	137	459	723	217	590	168	48	299	520	2529	1304
**rno-miR-455**	73	64	78	66	46	64	74	60	71	50	86	76
**rno-miR-465**	38	41	43	40	29	56	47	47	41	27	44	41
**rno-miR-466b**	56	54	42	58	52	46	53	66	49	53	75	56
**rno-miR-466d**	80	62	93	74	58	76	78	77	61	71	63	76
**rno-miR-501**	79	66	61	59	47	86	66	57	76	46	74	81
**rno-miR-539**	62	37	37	46	43	69	43	42	48	34	71	44
**rno-miR-543**	24	27	23	25	20	20	45	19	24	27	33	26

**Positive NF**	1.0	0.96	1.08	0.9	0.7	1.0	1.08	1.1	0.99	0.88	1.01	1.03

	4			3	8	3		9				
**rno-let-7b**	394	235	222	128	126	112	99	25	87	91	364	634
**rno-miR-122**	656	2745	1186	203	164	112	2411	56	5784	2007	1056	5238
**rno-miR-130a**	162	205	123	126	85	50	30	27	109	203	159	491
**rno-miR-150**	145	186	324	610	753	282	104	40	270	647	1790	1823
**rno-miR-20a+ rno-miR-20b-5p**	196	176	287	392	228	186	97	32	240	369	743	807
**rno-miR-22**	474	597	406	378	423	128	286	64	1042	798	1712	2123
**rno-miR-23a**	189	151	102	163	168	86	33	27	95	210	455	975
**rno-miR-25**	129	102	124	218	139	168	64	18	114	213	674	572
**rno-miR-27a**	115	145	130	179	178	56	40	29	93	238	438	636
**rno-miR-27b**	189	176	154	192	186	96	60	23	203	226	717	1142
**rno-miR-3571**	43	37	40	34	40	29	44	37	44	40	37	36
**rno-miR-3578**	51	39	32	46	40	42	47	43	32	57	51	36
**rno-miR-3579**	32	32	28	29	42	26	29	24	29	31	28	29
**rno-miR-3581**	35	37	32	27	40	34	32	45	30	30	39	34
**rno-miR-3585-3p**	34	33	39	37	37	27	40	35	36	38	41	46
**rno-miR-3589**	56	51	39	55	55	59	54	46	50	49	43	56
**rno-miR-3590-3p**	47	34	33	27	28	26	34	31	35	24	42	36
**rno-miR-3590-5p**	88	87	89	78	82	117	76	66	83	75	133	81
**rno-miR-3592**	42	39	40	35	29	32	39	35	37	36	40	32
**rno-miR-3593-5p**	110	102	88	92	112	105	73	107	93	84	105	85
**rno-miR-3594-5p**	132	102	111	111	126	103	112	91	106	97	119	107
**rno-miR-3595**	50	48	42	52	59	47	45	39	49	48	40	51

**rno-miR-3596c**	81	74	75	73	93	84	77	72	65	83	86	95
**rno-miR-376b-5p**	35	25	27	22	19	31	21	29	23	19.	20	31
**rno-miR-421**	109	82	95	92	69	73	83	75	71	106	94	84
**rno-miR-448**	31	20	20	19	19	20	21	29	26	24	19	30
**rno-miR-449c-3p**	155	152	136	158	153	142	166	124	149	138	159	157
**rno-miR-450a**	72	56	60	66	61	58	57	40	59	101	205	255
**rno-miR-451**	392	143	427	774	277	573	156	40	303	593	2494	1266
**rno-miR-455**	70	67	73	71	59	62	69	50	72	57	85	74
**rno-miR-465**	37	43	40	43	37	54	44	40	42	31	43	40
**rno-miR-466b**	54	56	39	62	66	45	49	55	50	60	74	54
**rno-miR-466d**	77	65	87	79	74	74	72	65	62	81	62	74
**rno-miR-501**	76	69	57	63	60	84	61	48	77	52	73	79
**rno-miR-539**	60	39	34	49	55	67	40	35	49	39	70	42
**rno-miR-543**	23	28	21	27	26	19	42	16	24	31	33	25

**GeoMean**	87	81	78	83	78	67	63	41	81	91	126	138
**Arithmetic mean of GeoMeans**	85											
**Total miRNA Normalisation Factor**	1.03	0.96	0.92	0.98	0.93	0.80	0.75	0.49	0.96	1.07	1.49	1.63

The raw count values were first divided by the positive control normalisation factor [Supplementary Table 3], the geometric mean (GeoMean) was calculated, and the arithmetic mean of these GeoMeans was taken. The Total RNA normalisation factor was then calculated by dividing the corresponding GeoMean by the arithmetic mean.

Supplementary Table 8. *In silico* recalculation: Background Thresholding plus NormFinder Normalisation recalculations for the 4 miRNA targets

**Control**	**Pregnant**

	R5	R8	R20	R22	R23	R24	R9	R11	R12	R14	R18	R19	
**miR-450a Raw values**	75	54	64	62	48	60	61	48	58	89	208	263	
**Positive Normalisation**	72.11	56.32	59.50	66.34	61.19	58.26	56.58	40.36	58.71	101.48	205.12	255.27	
**NormFinder**	104.68	81.23	87.23	81.58	83.78	153.10	170.64	283.68	74.98	91.21	84.03	79.75	**T-test**
**Mean**	104.68	81.23	87.23	81.58	83.78	153.10	170.64	283.68	74.98	91.21	84.03	79.75	0.403
**Mean+1SD**	104.68	81.23	87.23	81.58	83.78	153.10	170.64	283.68	74.98	91.21	84.03	79.75	0.403
**Mean+2SD**	104.68	81.23	87.23	81.58	83.78	153.10	170.64	283.68	74.98	91.21	84.03	79.75	0.403

	**Control**						**Pregnant**						
	**R5**	**R8**	**R20**	**R22**	**R23**	**R24**	**R9**	**R11**	**R12**	**R14**	**R18**	**R19**	
**miR-183 Raw values**	4	2	3	4	2	2	6	4	4	5	24	18	
**Positive Normalisation**	3.85	2.09	2.79	4.28	2.55	1.94	5.57	3.36	4.05	5.70	23.67	17.47	
**NormFinder**	5.58	3.01	4.09	5.26	3.49	5.10	16.78	23.64	5.17	5.12	9.70	5.46	**T-test**
**Mean**	13.13	11.25	13.38	10.13	8.50	11.75	16.78<br>(13.25)	23.64<br>(12.62)	12.38	9.38	14.38	14.25	0.12
**Mean+1SD**	18.43	16.64	16.88	15.09	13.07	15.44	21.73	23.64<br>(17.52)	17.27	14.39	18.41	19.69	0.07
**Mean+2SD**	23.73	22.03	20.38	20.06	17.63	19.14	30.21	23.64<br>(22.42)	22.17	19.40	22.44	25.14	0.09

	**Control**						**Pregnant**						
	**R5**	**R8**	**R20**	**R22**	**R23**	**R24**	**R9**	**R11**	**R12**	**R14**	**R18**	**R19**	
**miR-431 Raw values**	19	17	25	16	17	16	16	10	13	9	22	26	
**Positive Normalisation**	18.27	17.73	23.24	17.12	21.67	15.53	14.84	8.41	13.16	10.26	21.70	25.24	
**NormFinder**	26.52	25.57	34.07	21.05	29.67	40.83	44.76	59.10	16.81	9.22	8.89	7.88	**T-test**
**Mean**	26.52	25.57	34.07	21.05	29.67	40.83	44.76	59.10<br>(12.62)	16.81	9.38	14.38	14.25	0.73

	R5	R8	R20	R22	R23	R24	R9	R11	R12	R14	R18	R19	T-test
**Mean+1SD**	26.52	25.57	34.07	21.05	29.67	40.83	44.76 (21.73)	59.10 (17.52)	17.27	14.39	18.41	19.69	0.94
**Mean+2SD**	26.52 (23.73)	25.57 (22.03)	34.07	21.05 (20.06)	29.67 (17.63)	40.83 (19.14)	44.76 (30.21)	59.10 (22.42)	22.17	19.40	22.44	25.14	0.73

	**Control**						**Pregnant**						
	**R5**	**R8**	**R20**	**R22**	**R23**	**R24**	**R9**	**R11**	**R12**	**R14**	**R18**	**R19**	
**miR-196c Raw values**	19	13	26	15	19	31	12	10	17	13	25	22	
**Positive Normalisation**	18.27	13.56	24.17	16.05	24.22	30.10	11.13	8.41	17.21	14.82	24.65	21.35	
**NormFinder**	26.52	19.56	35.44	19.74	33.16	79.10	33.57	59.10	21.98	13.32	10.10	6.67	**T-test**
**Mean**	26.52	19.56	35.44	19.74	33.16	79.10	33.57 (13.25)	59.10 (12.62)	21.98	13.32	14.38	14.25	0.44
**Mean+1SD**	26.52	19.56 (16.64)	35.44	19.74 (15.09)	33.16	79.10	33.57 (21.73)	59.10 (17.52)	21.98 (17.27)	14.39	18.41	19.69	0.51
**Mean+2SD**	26.52 (23.73)	22.03	35.44	20.06	33.16	79.10	33.57 (30.21)	59.10 (22.42)	22.17	19.40	22.44	25.14	0.61

The raw values were first subjected to positive normalisation [Supplementary Table 3], followed by NormFinder normalisation [Supplementary Table 6]. The final values were then compared to the background levels [Supplementary Table 2], and if the value fell below the background value for that lane, the value was replaced by the background value. Red values highlight values that show discrepancies between the recalculation and nSolver, with nSolver values in brackets. Significance was tested using Welch's t-test. *P*-values highlighted in orange are those that were shown to be statistically significant (*P*-value < 0.05) by nSolver but were found to be non-significant upon recalculation.

Supplementary Table 9. *In silico* recalculation: Background Thresholding plus Total RNA Normalisation recalculations for the 4 miRNA targets

**Control**						**Pregnant**					
**R5**	**R8**	**R20**	**R22**	**R23**	**R24**	**R9**	**R11**	**R12**	**R14**	**R18**	**R19**

**miR-450a Raw Values**	75	54	64	62	48	60	61	48	58	89	208	263	
**Positive Normalisation**	72.11	56.32	59.37	52.13	48.59	68.42	60.16	46.59	53.93	95.22	265.18	255.35	
**Total miRNA Normalisation**	69.78	58.94	64.49	67.96	66.04	73.26	75.52	82.87	61.15	94.61	137.63	156.52	**T-test**
**Mean**	69.78	58.94	64.49	67.96	66.04	73.26	75.52	82.87	61.15	94.61	137.63	156.52	0.073
**Mean+1SD**	69.78	58.94	64.49	67.96	66.04	73.26	75.52	82.87	61.15	94.61	137.63	156.52	0.073
**Mean+2SD**	69.78	58.94	64.49	67.96	66.04	73.26	75.52	82.87	61.15	94.61	137.63	156.52	0.073

	**Control**						**Pregnant**						
	**R5**	**R8**	**R20**	**R22**	**R23**	**R24**	**R9**	**R11**	**R12**	**R14**	**R18**	**R19**	
**miR-183 Raw Values**	4	2	3	4	2	2	6	4	4	5	24	18	
**Positive Normalisation**	3.85	2.09	2.78	3.36	2.02	2.28	5.92	3.88	3.72	5.35	30.60	17.48	
**Total miRNA Normalisation**	3.72	2.18	3.02	4.38	2.75	2.44	7.43	6.91	4.22	5.32	15.88	10.71	**T-test**
**Mean**	13.13	11.25	13.38	10.13	8.50	11.75	13.25	12.63	12.38	9.38	15.88	14.25	0.199
**Mean+1SD**	18.43	16.64	16.88	15.09	13.07	15.44	21.73	17.52	17.27	14.39	18.41	19.69	0.107
**Mean+2SD**	23.73	22.03	20.38	20.06	17.63	19.14	30.21	22.42	22.17	19.40	22.44	25.14	0.111

	**Control**						**Pregnant**						
	**R5**	**R8**	**R20**	**R22**	**R23**	**R24**	**R9**	**R11**	**R12**	**R14**	**R18**	**R19**	
**miR-431 Raw Values**	19	17	25	16	17	16	16	10	13	9	22	26	
**Positive Normalisation**	18.27	17.73	23.19	13.45	17.21	18.24	15.78	9.71	12.09	9.63	28.05	25.24	
**Total miRNA Normalisation**	17.68	18.55	25.19	17.54	23.39	19.54	19.81	17.27	13.71	9.57	14.56	15.47	**T-test**
**Mean**	17.68	18.55	25.19	17.54	23.39	19.54	19.81	17.27<br>(12.62)	13.71	9.57<br>(9.38)	14.56	15.47	0.021
**Mean+1SD**	18.43	18.55	25.19	17.54	23.39	19.54	21.73	17.52	17.27	14.39	18.41	19.69	0.193
**Mean+2SD**	23.73	22.03	25.19	20.06	23.39<br>(17.63)	19.54<br>(19.14)	30.21	22.42	22.17	19.40	22.44	25.14	0.479

	Control						Pregnant						
	R5	R8	R20	R22	R23	R24	R9	R11	R12	R14	R18	R19	
**miR-196c Raw Values**	19	13	26	15	19	31	12	10	17	13	25	22	
**Positive Normalisation**	18.27	13.56	24.17	16.05	24.22	30.10	11.13	8.41	17.21	14.82	24.65	21.35	
**Total miRNA Normalisation**	17.68	14.19	26.26	20.92	32.92	32.23	13.97	14.96	19.51	14.73	12.80	13.09	**T-test**
**Mean**	17.68	14.19	26.26	20.92 (16.44)	32.92 (26.14)	32.23 (37.85)	13.97 (13.25)	14.96 (12.62)	19.51 (17.92)	14.73 (13.82)	14.38 (16.54)	14.25	0.039
**Mean+1SD**	18.43	16.64	26.26	20.92 (15.09)	32.92 (26.14)	32.23 (37.85)	21.73	17.52	19.51 (17.27)	14.73 (14.39)	18.41	19.69	0.094
**Mean+2SD**	23.73	22.03	26.26	20.92 (20.06)	32.92 (26.14)	32.23 (37.85)	30.21	22.42	22.17	19.40	22.44	25.14	0.321

The raw values were first subjected to positive normalisation [Supplementary Table 3], followed by total miRNA normalisation [Supplementary Table 7]. The final values were then compared to the background levels [Supplementary Table 2], and of the value fell below the background value for that lane, the value was replaced by the background value. Red values highlight values that show discrepancies between the recalculation and nSolver, with nSolver values in brackets. Significance was tested using Welch's t-test. *P*-values highlighted in green are those that were found to be statistically significant (*P*-value < 0.05) by nCounter and agreed by the recalculation.

	Control						Pregnant						
	R5	R8	R20	R22	R23	R24	R9	R11	R12	R14	R18	R19	
**miR-450a Raw Values**	75	54	64	62	48	60	61	48	58	89	208	263	
**Subtraction (Mean)**	61.88	42.75	50.63	51.88	39.50	48.25	47.75	35.38	45.63	79.63	193.63	248.75	
**Positive Normalisation**	59.49	44.59	47.07	55.50	50.36	46.85	44.29	29.74	46.18	90.79	190.95	241.43	**T-test**

**NormFinder**	86.36 (89.2)	64.31 (66.49)	69.00 (71.6)	68.26 (68.91)	68.94 (70.1)	123.12 (133.86)	133.57 (154)	209.06 (291.37)	58.98 (61.45)	81.60 (80.63)	78.22 (75.81)	75.42 (72.54)	0.329
**Subtraction (Mean+1SD)**	56.57	37.36	47.12	46.91	34.93	44.56	39.27	30.48	40.73	74.61	189.59	243.31	
**Positive Normalisation**	54.39	38.96	43.81	50.19	44.54	43.26	36.43	25.63	41.22	85.08	186.97	236.15	T-test
**NormFinder**	78.96 (82.57)	56.20 (59.39)	64.22 (66.59)	61.72 (62.86)	60.97 (63.07)	113.69 (126.38)	109.85 (143.35)	180.12 (304.16)	52.65 (55.77)	76.47 (75.45)	76.59 (72.71)	73.77 (69.51)	0.314
**Subtraction (Mean+2SD)**	51.27	31.97	43.62	41.94	30.37	40.86	30.79	25.58	35.83	69.60	185.56	237.86	
**Positive Normalisation**	49.29	33.34	40.56	44.87	38.71	39.67	28.56	21.51	36.27	79.36	182.99	230.87	T-test
**NormFinder**	71.56 (75.79)	48.09 (52.10)	59.45 (61.55)	55.18 (56.73)	53.00 (58.89)	104.27 (118.66)	86.12 (137.79)	151.18 (340.06)	46.32 (49.98)	71.33 (70.22)	74.96 (69.56)	72.12 (66.43)	0.305

	**Control**						**Pregnant**						
	**R5**	**R8**	**R20**	**R22**	**R23**	**R24**	**R9**	**R11**	**R12**	**R14**	**R18**	**R19**	
**miR-183 Raw Values**	4	2	3	4	2	2	6	4	4	5	24	18	
**Subtraction (Mean)**	1.00	1.00	1.00	1.00	1.00	1.00	1.00	1.00	1.00	1.00	9.63	3.75	
**Positive Normalisation**	0.96	1.04	0.93	1.07	1.27	0.97	0.93	0.84	1.01	1.14	9.49	3.64	**T-test**
**NormFinder**	1.40 (1.44)	1.50 (1.56)	1.36 (1.41)	1.32 (1.33)	1.75 (1.77)	2.55 (2.77)	2.80 (3.23)	5.91 (8.24)	1.29 (1.35)	1.02 (1.01)	3.89 (3.77)	1.14 (1.09)	0.258
**Subtraction (Mean+1SD)**	1	1	1	1	1	1	1	1	1	1	5.59	1	

**Positive Normalisation**	0.96	1.04	0.93	1.07	1.27	0.97	0.93	0.84	1.01	1.14	5.51	0.97	T-test
**NormFinder**	1.40<br>(1.46)	1.50<br>(1.59)	1.36<br>(1.41)	1.32<br>(1.34)	1.75<br>(1.81)	2.55<br>(2.84)	2.80<br>(3.7)	5.91<br>(9.98)	1.29<br>(1.37)	1.02<br>(1.01)	2.26<br>(2.14)	0.30<br>(1)	0.490
**Subtraction (Mean+2SD)**	1.00	1.00	1.00	1.00	1.00	1.00	1.00	1.00	1.00	1.00	1.56	1.00	
**Positive Normalisation**	0.96	1.04	0.93	1.07	1.27	0.97	0.93	0.84	1.01	1.14	1.54	0.97	T-test
**NormFinder**	1.40<br>(1.48)	1.50<br>(1.63)	1.36<br>(1.41)	1.32<br>(1.35)	1.75<br>(1.84)	2.55<br>(2.9)	2.80<br>(4.48)	5.91<br>(13.29)	1.29<br>(1.39)	1.02<br>(1.01)	0.63<br>(1)	0.30<br>(1)	0.708

Supplementary Table 10. *In silico* recalculation: Background Subtraction plus NormFinder Normalisation recalculations for the 4 miRNA targets

	**Control**						**Pregnant**						
	**R5**	**R8**	**R20**	**R22**	**R23**	**R24**	**R9**	**R11**	**R12**	**R14**	**R18**	**R19**	
**miR-431 Raw Values**	19	17	25	16	17	16	16	10	13	9	22	26	
**Subtraction (Mean)**	5.88	5.75	11.63	5.88	8.50	4.25	2.75	1	1	1	7.63	11.75	
**Positive Normalisation**	5.65	6.00	10.81	6.29	10.84	4.13	2.55	0.84	1.01	1.14	7.52	11.40	**T-test**
**NormFinder**	8.20<br>(8.47)	8.65<br>(8.94)	15.84<br>(16.44)	7.73<br>(7.8)	14.84<br>(15.09)	10.84<br>(11.79)	7.69<br>(8.87)	5.91<br>(8.24)	1.29<br>(1.35)	1.02<br>(1.01)	3.08<br>(2.99)	3.56<br>(3.43)	0.003
**Subtraction (Mean+1SD)**	1	1	8.12	1	3.93	1	1	1	1	1	3.59	6.31	
**Positive Normalisation**	0.96	1.04	7.55	1.07	5.01	0.97	0.93	0.84	1.01	1.14	3.54	6.12	**T-test**
**NormFinder**	1.40<br>(1.46)	1.50<br>(1.59)	11.07<br>(11.49)	1.32<br>(1.34)	6.86<br>(7.1)	2.55<br>(2.84)	2.80<br>(3.7)	5.91<br>(9.98)	1.29<br>(1.37)	1.02<br>(1.01)	1.45<br>(1.38)	1.91<br>(1.8)	0.371
**Subtraction (Mean+2SD)**	1	1	4.62	1	1	1	1	1	1	1	1	1	
**Positive Normalisation**	0.96	1.04	4.30	1.07	1.27	0.97	0.93	0.84	1.01	1.14	0.99	0.97	**T-test**
**NormFinder**	1.40<br>(1.48)	1.50<br>(1.63)	6.30<br>(6.52)	1.32<br>(1.35)	1.75<br>(1.84)	2.55<br>(2.9)	2.80<br>(4.48)	5.91<br>(13.29)	1.29<br>(1.39)	1.02<br>(1.01)	0.40<br>(1)	0.30<br>(1)	0.672

	Control						Pregnant						
	R5	R8	R20	R22	R23	R24	R9	R11	R12	R14	R18	R19	
miR-196c Raw Values	19	13	26	15	19	31	12	10	17	13	25	22	
Subtraction (Mean)	5.88	1.75	12.63	4.88	10.50	19.25	1.00	1	5	4	10.63	7.75	
Positive Normalisation	5.65	1.83	11.74	5.22	13.39	18.69	0.93	0.84	4.68	4.13	10.48	7.52	T-test
NormFinder	8.20 (8.47)	2.63 (2.72)	17.21 (17.86)	6.41 (6.48)	18.33 (18.63)	49.12 (53.41)	2.80 (3.23)	5.91 (8.24)	5.98 (6.23)	3.72 (3.67)	4.29 (4.16)	2.35 (2.26)	0.123

Subtraction (Mean±1SD)	1	1	9.12	1	5.93	16	1	1	1	1	6.59	2.31	
Positive Normalisation	0.96	1.04	8.48	1.07	7.56	15.10	0.93	0.84	1.01	1.14	6.50	2.24	T-test
NormFinder	1.40 (1.46)	1.50 (1.59)	12.43 (12.89)	1.32 (1.34)	10.36 (10.71)	39.69 (44.12)	2.80 (3.70)	5.91 (9.98)	1.29 (1.37)	1.02 (1.01)	2.66 (2.53)	0.70 (1)	0.211

Subtraction (Mean±2SD)	1	1	5.62	1	1	12	1	1	1	1	3	1	
Positive Normalisation	0.96	1.04	5.23	1.07	1.74	11.52	0.93	0.84	1.01	1.14	2.52	0.97	T-test
NormFinder	1.40 (1.48)	1.50 (1.63)	7.66 (7.93)	1.32 (1.35)	2.38 (2.51)	30.27 (34.45)	2.80 (4.48)	5.91 (13.29)	1.29 (1.39)	1.02 (1.01)	1.03 (1)	0.30 (1)	0.307

The raw values were first subjected to background subtraction [Supplementary Table 2], followed by positive normalisation [Supplementary Table 3] and then NormFinder normalisation[Supplementary Table 6]. Red values highlight values that show discrepancies between the recalculation and nCounter, with final nSolver values in brackets. Significance was tested using Welch's t-test. *P*-values highlighted in green are those that were found to be statistically significant (*P*-value < 0.05) by nCounter and agreed by the recalculation.

	Control						Pregnant						
	R5	R8	R20	R22	R23	R24	R9	R11	R12	R14	R18	R19	
miR-450a Raw Values	75	54	64	62	48	60	61	48	58	89	208	263	
Subtraction (Mean)	61.88	42.75	50.63	51.88	39.50	48.25	47.75	35.38	45.63	79.63	193.63	248.75	
Positive Normalisation	59.49	44.59	47.07	55.50	50.36	46.85	44.29	29.74	46.18	90.79	190.95	241.43	T-test

**Total miRNA Normalisation**	57.57 (56.08)	46.66 (46.05)	51.13 (52.32)	72.35 (55.17)	68.45 (52.84)	50.16 (59.45)	55.60 (61.53)	52.91 (67.61)	52.37 (48.66)	90.21 (81.62)	99.10 (130.58)	147.99 (147.68)	0.167
**Subtraction (Mean + 1SD)**	56.57	37.36	47.12	46.91	34.93	44.56	39.27	30.48	40.73	74.61	189.59	243.31	
**Positive Normalisation**	54.39	38.96	43.81	50.19	44.54	43.26	36.43	25.63	41.22	85.08	186.97	236.15	**T-test**
**Total miRNA Normalisation**	52.64 (49.47)	40.77 (40.67)	47.59 (46.11)	65.42 (49.93)	60.53 (48.2)	46.32 (52.56)	45.73 (60.1)	45.58 (61.9)	46.75 (42.95)	84.53 (77.31)	97.04 (125.76)	144.75 (141.53)	0.186
**Subtraction (Mean + 2SD)**	51.27	31.97	43.62	41.94	30.37	40.86	30.79	25.58	35.83	69.60	185.56	237.86	
**Positive Normalisation**	49.29	33.34	40.56	44.87	38.71	39.67	28.56	21.51	36.27	79.36	182.99	230.87	**T-test**
**Total miRNA Normalisation**	47.70 (43.56)	34.89 (35.22)	44.05 (40.33)	58.49 (45.98)	52.62 (41.91)	42.48 (46.45)	35.85 (56.85)	38.26 (54.12)	41.13 (38.94)	78.85 (75.11)	94.97 (112.03)	141.51 (140.19)	0.207

Supplementary Table 11. *In silico* recalculation: Background Subtraction plus Total RNA Normalisation recalculations for the 4 miRNA targets

	**Control**						**Pregnant**						
	**R5**	**R8**	**R20**	**R22**	**R23**	**R24**	**R9**	**R11**	**R12**	**R14**	**R18**	**R19**	
**miR-183 Raw Values**	4	2	3	4	2	2	6	4	4	5	24	18	
**Subtraction (Mean)**	1	1	1	1	1	1	1	1	1	1	9.63	3.75	
**Positive Normalisation**	0.96	1.04	0.93	1.07	1.27	0.97	0.93	0.84	1.01	1.14	9.49	3.64	**T-test**
**Total miRNA Normalisation**	0.93 (1)	1.1	1.01 (1.03)	1.39 (1.06)	1.73 (1.34)	1.04 (1.23)	1.16 (1.29)	1.50 (1.91)	1.15 (1.07)	1.13 (1.03)	4.93 (6.49)	2.23	0.240
**Subtraction (Mean + 1SD)**	1	1	1	1	1	1	1	1	1	1	5.59	1	

**Positive Normalisation**	0.96	1.04	0.93	1.07	1.27	0.97	0.93	0.84	1.01	1.14	5.51	0.97	**T-test**
**Total miRNA Normalisation**	0.93 (1)	1.1	1.01 (1)	1.39 (1.06)	1.73 (1.38)	1.04 (1.18)	1.16 (1.53)	1.50 (2.03)	1.15 (1.05)	1.13 (1.04)	2.86 (3.71)	0.59 (1)	0.576

**Subtraction (Mean + 2SD)**	1	1	1	1	1	1	1	1	1	1	1.56	1	
**Positive Normalisation**	0.96	1.04	0.93	1.07	1.27	0.97	0.93	0.84	1.01	1.14	1.54	0.97	**T-test**
**Total miRNA Normalisation**	0.93 (1)	1.1	1.01 (1)	1.39 (1.1)	1.73 (1.38)	1.04 (1.14)	1.16 (1.85)	1.50 (2.12)	1.15 (1.09)	1.13 (1.08)	0.80 (1)	0.59 (1)	0.440

	**Control**						**Pregnant**						
	**R5**	**R8**	**R20**	**R22**	**R23**	**R24**	**R9**	**R11**	**R12**	**R14**	**R18**	**R19**	
**miR-431 Raw Values**	19	17	25	16	17	16	16	10	13	9	22	26	
**Subtraction (Mean)**	5.88	5.75	11.63	5.88	8.50	4.25	2.75	1	1	1	7.63	11.75	
**Positive Normalisation**	5.65	6.00	10.81	6.29	10.84	4.13	2.55	0.84	1.01	1.14	7.52	11.40	**T-test**
**Total miRNA Normalisation**	5.47 (5.32)	6.28 (6.19)	11.74 (12.01)	8.19 (6.25)	14.73 (11.37)	4.42 (5.24)	3.20 (3.54)	1.50 (1.91)	1.15 (1.07)	1.13 (1.03)	3.90 (5.14)	6.99 (6.98)	0.019

**Subtraction (Mean + 1SD)**	1	1	8.12	1	3.93	1	1	1	1	1	3.59	6.31	
**Positive Normalisation**	0.96	1.04	7.55	1.07	5.01	0.97	0.93	0.84	1.01	1.14	3.54	6.12	**T-test**
**Total miRNA Normalisation**	0.93 (1)	1.1	8.20 (7.95)	1.39 (1.06	6.82 (5.43)	1.04 (1.18)	1.16 (1.53)	1.50 (2.03)	1.15 (1.05)	1.13 (1.04)	1.84 (2.38)	3.75 (3.67)	0.336

**Subtraction (Mean + 2SD)**	1	1	4.62	1	1	1	1	1	1	1	1	1	
**Positive Normalisation**	0.96	1.04	4.30	1.07	1.27	0.97	0.93	0.84	1.01	1.14	0.99	0.97	**T-test**
**Total miRNA Normalisation**	0.93 (1)	1.1	4.67 (4.27)	1.39 (1.1)	1.73 (1.38)	1.04 (1.14)	1.16 (1.85)	1.50 (2.12)	1.15 (1.09)	1.13 (1.08)	0.51 (1)	0.59 (1)	0.235

	Control						Pregnant						
	R5	R8	R20	R22	R23	R24	R9	R11	R12	R14	R18	R19	
**miR-196c Raw Values**	19	13	26	15	19	31	12	10	17	13	25	22	
**Subtraction (Mean)**	5.88	1.75	12.63	4.88	10.50	19.25	1.00	1	5	4	10.63	7.75	
**Positive Normalisation**	5.65	1.83	11.74	5.22	13.39	18.69	0.93	0.84	4.68	4.13	10.48	7.52	**T-test**
**Total miRNA Normalisation**	5.47 (5.32)	1.91 (1.89)	12.75 (13.05)	6.80 (5.18)	18.19 (14.04)	20.01 (23.72)	1.16 (1.29)	1.50 (1.91)	5.31 (4.93)	4.11 (3.72)	5.44 (7.17)	4.61	0.061
**Subtraction (Mean±1SD)**	1	1	9.12	1	5.93	16	1	1	1	1	6.59	2.31	
**Positive Normalisation**	0.96	1.04	8.48	1.07	7.56	15.10	0.93	0.84	1.01	1.14	6.50	2.24	**T-test**
**Total miRNA Normalisation**	0.93 (1)	1.09	9.21 (8.93)	1.39 (1.06)	10.28 (8.19)	16.17 (18.35)	1.16 (1.53)	1.50 (2.03)	1.15 (1.05)	1.13 (1.04)	3.37 (4.37)	1.37 (1.34)	0.118
**Subtraction (Mean±2SD)**	1	1	5.62	1	1	12	1	1	1	1	3	1	
**Positive Normalisation**	0.96	1.04	5.23	1.07	1.74	11.52	0.93	0.84	1.01	1.14	2.52	0.97	**T-test**
**Total miRNA Normalisation**	0.93 (1)	1.09 (1.10)	5.68 (5.20)	1.39 (1.10)	2.37 (1.89)	12.33 (13.54)	1.16 (1.85)	1.50 (2.12)	1.15 (1.09)	1.13 (1.08)	1.31 (1.54)	0.59 (1)	0.182

The raw values were first subjected to background subtraction [Supplementary Table 2], followed by positive normalisation [Supplementary Table 3] and then total miRNA normalisation [Supplementary Table 7]. Red values highlight values that show discrepancies between the recalculation and nCounter, with final nSolver values in brackets. Significance was tested using Welch's t-test. *P*-values highlighted in orange are those that were found to be statistically significant (*P*-value < 0.05) by nCounter but were not replicated in the recalculation. *P*-values highlighted in green are those that were found to be statistically significant (*P*-value < 0.05) by nCounter and agreed by the recalculation.

Supplementary Table 12. ***In silico*** **recalculation: No Background Subtraction plus NormFinder Normalisation recalculations for the 4 miRNA targets**

	Control						Pregnant					
	R5	R8	R20	R22	R23	R24	R9	R11	R12	R14	R18	R19
**miR-450a Raw Values**	75	54	64	62	48	60	61	48	58	89	208	263

Positive Normalisation	72.11	56.32	59.50	66.34	61.19	58.26	56.58	40.36	58.71	101.48	205.12	255.27	T-test
NormFinder	104.68	81.23	87.23	81.58	83.78	153.10	170.64	283.68	74.98	91.21	84.03	79.75	0.77

	Control						Pregnant						
	**R5**	**R8**	**R20**	**R22**	**R23**	**R24**	**R9**	**R11**	**R12**	**R14**	**R18**	**R19**	
**miR-183 Raw Values**	4	2	3	4	2	2	6	4	4	5	24	18	
Positive Normalisation	3.85	2.09	2.79	4.28	2.55	1.94	5.57	3.36	4.05	5.70	23.67	17.47	**T-test**
NormFinder	5.58	3.01	4.09	5.26	3.49	5.10	16.78	23.64	5.17	5.12	9.70	5.46	0.045

	Control						Pregnant						
	**R5**	**R8**	**R20**	**R22**	**R23**	**R24**	**R9**	**R11**	**R12**	**R14**	**R18**	**R19**	
**miR-431 Raw Values**	19	17	25	16	17	16	16	10	13	9	22	26	
Positive Normalisation	18.27	17.73	23.24	17.12	21.67	15.53	14.84	8.41	13.16	10.26	21.70	25.24	**T-test**
NormFinder	26.52	25.57	34.07	21.05	29.67	40.83	44.76	59.10	16.81	9.22	8.89	7.88	0.30

	Control						Pregnant						
	**R5**	**R8**	**R20**	**R22**	**R23**	**R24**	**R9**	**R11**	**R12**	**R14**	**R18**	**R19**	
**miR-196c Raw Values**	19	13	26	15	19	31	12	10	17	13	25	22	
Positive Normalisation	18.27	13.56	24.17	16.05	24.22	30.10	11.13	8.41	17.21	14.82	24.65	21.35	**T-test**
NormFinder	26.52	19.56	35.44	19.74	33.16	79.10	33.57	59.10	21.98	13.32	10.10	6.67	0.37

The raw values were first subjected to positive normalisation [Supplementary Table 3] followed by NormFinder normalisation[Supplementary Table 6]. Significance was tested using Welch's t-test. *P*-values highlighted in green are those that were found to be statistically significant (*P*-value < 0.05) by nCounter and agreed by the recalculation.

Supplementary Table 13. *In silico* recalculation: No Background Subtraction plus Total RNA Normalisation recalculations for the 4 miRNA targets.

Control	Pregnant

	R5	R8	R20	R22	R23	R24	R9	R11	R12	R14	R18	R19	
**miR-450a Raw Values**	75	54	64	62	48	60	61	48	58	89	208	263	
**Positive Normalisation**	72.11	56.32	59.37	52.13	48.59	68.42	60.16	46.59	53.93	95.22	265.18	255.35	**T-test**
**Total miRNA Normalisation**	69.78	58.94	64.49	67.96	66.04	73.26	75.52	82.87	61.15	94.61	137.63	156.52	0.09

	**Control**						**Pregnant**						
	R5	R8	R20	R22	R23	R24	R9	R11	R12	R14	R18	R19	
**miR-183 Raw Values**	4	2	3	4	2	2	6	4	4	5	24	18	
**Positive Normalisation**	3.85	2.09	2.78	3.36	2.02	2.28	5.92	3.88	3.72	5.35	30.60	17.48	**T-test**
**Total miRNA Normalisation**	3.72	2.18	3.02	4.38	2.75	2.44	7.43	6.91	4.22	5.32	15.88	10.71	0.03

	**Control**						**Pregnant**						
	R5	R8	R20	R22	R23	R24	R9	R11	R12	R14	R18	R19	
**miR-431 Raw Values**	19	17	25	16	17	16	16	10	13	9	22	26	
**Positive Normalisation**	18.27	17.73	23.19	13.45	17.21	18.24	15.78	9.71	12.09	9.63	28.05	25.24	**T-test**
**Total miRNA Normalisation**	17.68	18.55	25.19	17.54	23.39	19.54	19.81	17.27	13.71	9.57	14.56	15.47	0.02

	**Control**						**Pregnant**						
	R5	R8	R20	R22	R23	R24	R9	R11	R12	R14	R18	R19	
**miR-196ca Raw Values**	19	13	26	15	19	31	12	10	17	13	25	22	
**Positive Normalisation**	18.27	13.56	24.17	16.05	24.22	30.10	11.13	8.41	17.21	14.82	24.65	21.35	**T-test**
**Total miRNA Normalisation**	17.68	14.19	26.26	20.92 (16.44)	32.92 (26.14)	32.23 (37.85)	13.97 (14.86)	14.96 (17.27)	19.51 (17.92)	14.73 (13.82)	12.80 (16.54)	13.09	0.03

The raw values were first subjected to positive normalisation [Supplementary Table 3] followed by total miRNA normalisation[Supplementary Table 7]. Significance was tested using Welch's t-test. *P*-values highlighted in green are those that were found to be statistically significant (*P*-value < 0.05) by nCounter and agreed by the recalculation. *P*-values highlighted in blue are those that were found to be significant

according to the recalculation but were not identified as a significant change by nSolver.
